# Cracking effects in squashable and stretchable thin metal films on PDMS for flexible microsystems and electronics

**DOI:** 10.1038/s41598-018-27798-z

**Published:** 2018-06-22

**Authors:** Tiffany Baëtens, Emiliano Pallecchi, Vincent Thomy, Steve Arscott

**Affiliations:** Institut d’Electronique, de Microélectronique et de Nanotechnologie (IEMN), CNRS, The University of Lille, Cité Scientifique, 59652 Villeneuve d’Ascq, France

## Abstract

Here, we study cracking of nanometre and sub-nanometre-thick metal lines (titanium, nickel, chromium, and gold) evaporated onto commercial polydimethylsiloxane (PDMS) substrates. Mechanical and electromechanical testing reveals potentially technologically useful effects by harnessing cracking. When the thin film metal lines are subjected to uniaxial longitudinal stretching, strain-induced cracks develop in the film. The regularity of the cracking is seen to depend on the applied longitudinal strain and film thickness—the findings suggest ordering and the possibility of creating metal mesas on flexible substrates without the necessity of lithography and etching. When the metal lines are aligned transversally to the direction of the applied strain, a Poisson effect-induced electrical ‘self-healing’ can be observed in the films. The Poisson effect causes process-induced cracks to short circuit, resulting in the lines being electrically conducting up to very high strains (~40%). Finally, cracking results in the observation of an enhanced transversal gauge factor which is ~50 times larger than the geometric gauge factor for continuous metal films—suggesting the possibility of high-sensitivity thin-film metal strain gauge flexible technology working up to high strains.

## Introduction

Understanding the behaviour of materials under high mechanical stresses and strains is the key to being able to optimise processes for the manufacture of original and robust flexible^1^, stretchable^2^, and squashable electronic systems—this approach will no doubt have a major impact in a multitude of applications including *inter alia* wearable electronics^3–5^, displays^6,7^ soft robotics^8,9^, and biomedical fields^10–12^. An important part of any electronic system is that of reliable, robust conducting interconnections which link the different devices and components on the integrated chip. This is all the more critical for flexible, stretchable and squashable systems—where parts, including interconnections, can be exposed to high mechanical strains causing stresses to be generated. Such stresses can result in mechanical rupture when the ultimate tensile or compressive strength of the material is exceeded—ultimately leading to device and system failure. In the quest for robust interconnections on soft substrates, several materials and processes are currently being investigated—these include: thin films^13–26^, nanowires^27^, nanomembranes^28^, two dimensional materials^29^, one dimensional materials^30^, polymeric conductors^31^, carbon coatings^32^, and liquid metals^33,34^. Amongst these, thin films have the advantage that they are well known in the microelectronics industry and compatible—or at least *in principle* can be made compatible—with standard micro and nano fabrication planar processes^35,36^. However, cracking is often observed in such films—especially at the high mechanical strains required by flexible, and more so, stretchable applications. It is important to note that it is now becoming apparent that the specific type of mechanical solicitation determines the observed electromechanical behaviour^37^. In this context, the present article addresses some of the issues concerned in the quest for robust, thin film interconnections for squashable and stretchable electronics using thin metal films. More specifically, these issues include: type of adhesion metal best adapted to stretchable and squashable systems, the impact of metal thickness in the nanometre and sub-nanometre domain on performance, and the importance of system architecture. Finally, the work here can also be set into a more general context since cracking is a universal phenomenon which occurs in materials on the macroscopic scale^38,39^, the micrometre scale^40–43^, and the nanometre scale^44,45^.

## Results and Discussion

### Methodology of the study

**Figure 1:**
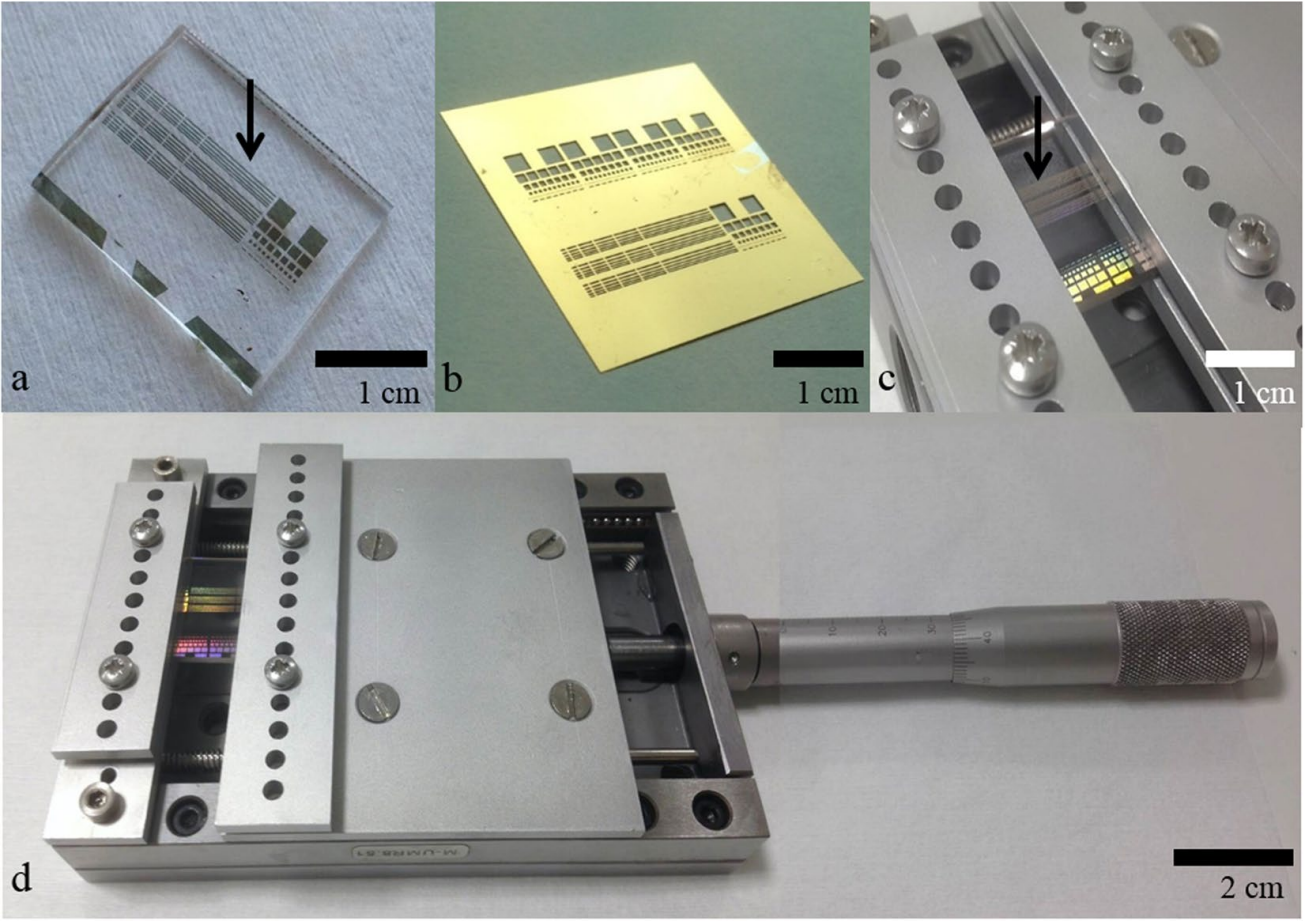
Fabrication and mechanical testing the metallized polydimethylsiloxane (PDMS). (**a**) Thin film metal features are thermally evaporated onto a PDMS support. **(b)** A thin physical ‘shadow’ mask used for the patterning of the metallic features. **(c)** The metallized PDMS sample is mounted into a simple testing machine which applies strain to the sample. (**d**) The testing machine is composed of a modified high-precision displacement-sliding table.

**Figure 2:**
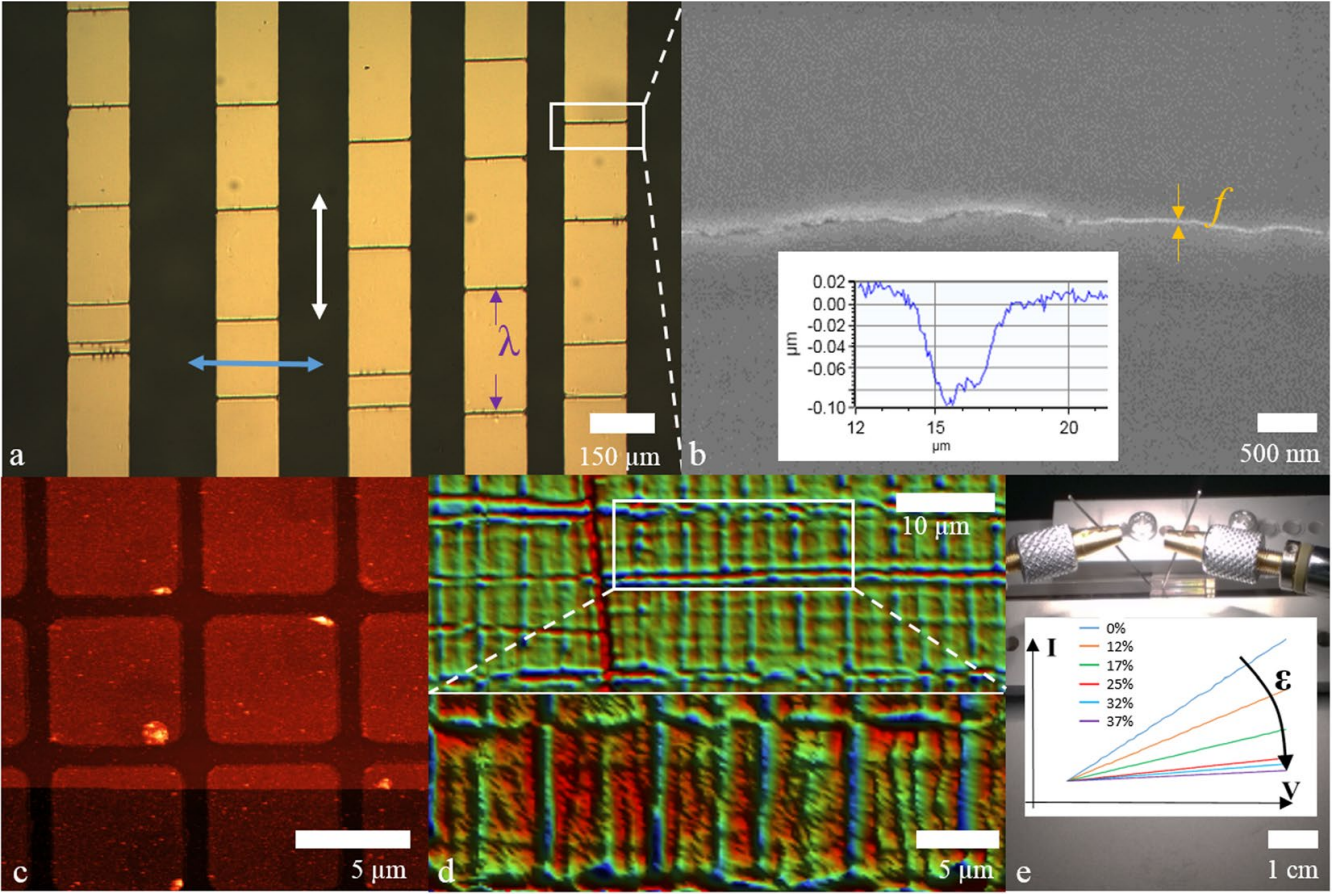
Measurement techniques used in the study. (**a**) Optical microscopy. The white and blue arrows indicate the strain directions relative to the long lengths of the wires used in the study. The characteristic crack spacing is indicated by *λ*. (**b**) Scanning electron microscopy (SEM)—the crack width *f* is visible. The inset shows a depth profile of a crack. (**c**) Atomic force microscopy (AFM) was used to measure the evaporated film thickness and uniformity. (**d**) Optical interference microscopy was used to produce non-contact, large surface tomography of cracking and buckling. (**e**) Piezoresistance characterization via current-voltage (*IV*) measurements using a probe station. The inset shows how the *IV* changes with applied strain.

Figures 1 and 2 illustrate the general methodology of the study undertaken here.

Figure 1 shows the fabrication methods and strain testing of the metallized polydimethylsiloxane (PDMS) elastomer—see Methods for a detailed explanation of the preparation of the PDMS samples. Figure 1a shows a PDMS sample which has been patterned with thermally evaporated thin film metal features (indicated by the black arrow in Fig. 1a) using the physical ‘shadow’ mask containing holes shown in Fig. 1b. The PDMS substrates are moulded to have a thickness of 1.7 mm and subsequently diced—prior to evaporation—to be 2 cm wide and 4 cm long. Following a previous study^44^, the studied adhesion metal thicknesses here are <5 nm in an effort to minimise process-associated film cracking. Concerning the gold thickness, previous work suggests that 10–50 nm of metal, mostly gold, is a suitable choice for flexible electronics.

Details concerning the evaporated metal features’ specific deposition parameters—type, lengths, widths, and thicknesses—can be found at the end of the article (see Methods). The metallized PDMS samples are mounted into an in-house mechanical strain applicator which is a modified high-precision displacement sliding table—shown in Fig. 1c—capable of applying uniaxial strain to the metallized PDMS samples. The black arrow in Fig. 1c indicates the metal lines under uniaxial tensile stress. The strain resolution of the device is 0.1%, which is largely sufficient for the strains to be applied to the sample—up to 50%. The strain is increased by manually turning the precision displacement table shown in Fig. 1d.

Figure 2 shows the various measurement techniques used in the study—details can be found at the end of the paper (see Methods). These include optical microscopy—Fig. 2a, scanning electron microscopy (SEM)—Fig. 2b, atomic force microscopy (AFM)—Fig. 2c, optical interference microscopy—Fig. 2d, and piezoresistance current-voltage (*IV*) measurements—Fig. 2e.

The number of cracks (per unit distance) and the crack spacing of the thin metal films were observed using optical microscopy—see Fig. 2a. The local cracking was studied using scanning electron microscopy—see Fig. 2b. The thickness, roughness, and uniformity of the evaporated metal films were measured using atomic force microscopy (AFM)—see Fig. 2c (see Supplementary Table S1). The large surface topography of the samples under mechanical strain was studied using optical interference microscopy—see Fig. 2d. The piezoresistance of the thin film metal lines was measured as a function of applied strain using a commercial probe station—see Fig. 2e. The characteristic crack spacing *λ* is indicated on Fig. 2a and the crack width *f* is indicated in Fig. 2b. Throughout the study, we use the experimental *average* characteristic crack spacing to be ${\lambda }_{a}$ and the *average* crack length to be ${f}_{a}$—as these values are gathered statistically using a number of metallized lines on several samples (see Methods). The cracking of the films was recorded on samples at zero-strain—we refer to this as spontaneous *process-induced* cracking (PIC). During the application of strain using the strain applicator cracking appears in the films—we refer to this as externally imposed *strain-induced* cracking (SIC).

### Process-induced cracking (PIC)

The first part of the study is concerned with spontaneous cracking of thin metal films evaporated onto PDMS which incurs due to the residual tensile stress in the thin film resulting from the evaporation process^46^—we refer to this here as ‘process-induced cracking’ (PIC). Four types of thin films were evaporated onto PDMS substrates to study this: titanium/gold, nickel/gold, chromium/gold, and gold (see Supplementary Figure S1 for details). The reason for this is that titanium, nickel, and chromium are three common ‘adhesion metals’ often used in micro and nano technologies, and microelectronics^35,36^. The lateral dimensions of the evaporated lines are 7.95 mm by 150 µm.

It was observed that there was no PIC when using evaporated gold and nickel/gold. However, gold did not adhere well to PDMS surface even after an optimised^44^ oxygen plasma treatment which all PDMS samples were subjected to prior to evaporation of the metal films—this is well known^47^. Some PIC was observed when using titanium as the adhesion layer—although it should be noted that many lines were crack-free. In contrast, when using chromium films, PIC strongly depends on the thickness. The process-induced stresses in chromium thin films are well known to be much higher—at least when using rigid substrates^48^. However, the lines are well defined with the PIC perpendicular to the line edges^42^. The result of the cracking is the formation of isolated rectangular metallic *mesa* features along the lines—which suggests a certain degree of ordering^44^ due to a characteristic crack spacing^49^. This can be contrasted with the ordering in such systems due to buckling^15^. A comparison of different thickness of chromium as adhesion film completes the study about PIC.

**Figure 3:**
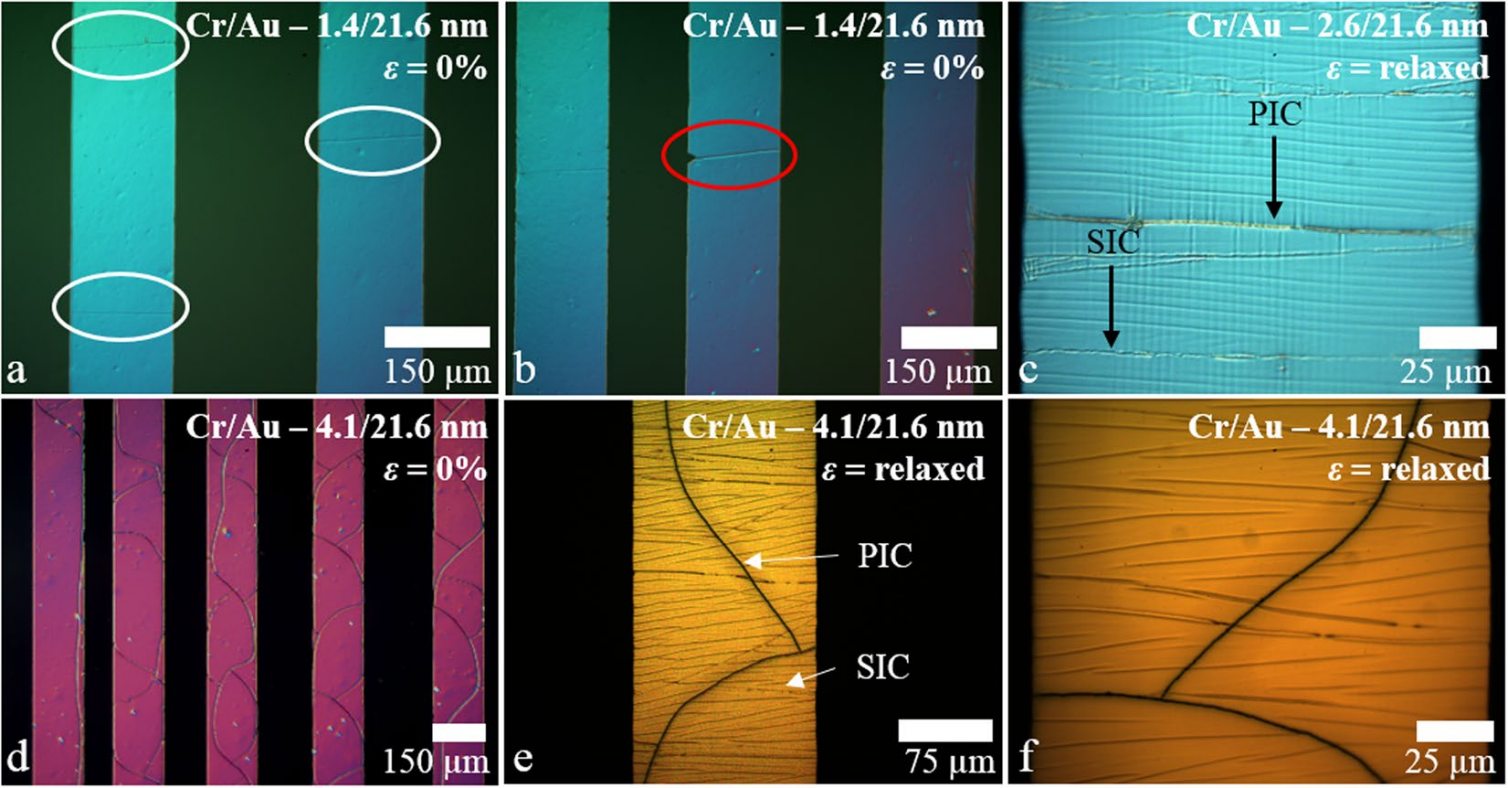
The effect of film thickness on process-induced cracking of thin metal films evaporated onto PDMS. (**a**) A chromium/gold (1.4/21.6 nm) evaporated line results in PIC perpendicular to the line edge—indicated by the white ellipses. (**b**) A PIC associated with a large edge defect. (**c**) Zoom on a chromium/gold (2.6/21.6 nm) line showing the difference between PIC and external strain-induced cracking (SIC). (**d**) A chromium/gold (4.1/21.6 nm) evaporated line results in a curved or ‘wavy’ PIC^50^. (**e**) and (**f**) Zooms on the chromium/gold (4.1/21.6 nm) metallization showing the difference between PIC and external strain-induced cracking (SIC) after external straining in a relaxed state. The lines are 150 µm wide.

Figure 3 shows optical microscopy photographs—taken using polarized light—of metallized evaporated chromium/gold lines having different chromium thicknesses on PDMS.

The evaporation of different chromium thickness films onto PDMS demonstrates two different types of PIC observed in the study. When the chromium thickness is relatively small (1.4 nm) the PIC is always perpendicular to the line edges. Increasing the chromium thickness to 4.1 nm results in the PIC becoming curved or ‘wavy’—this has previously been predicted^50^. Note that some external post-process strain-induced cracking (SIC) is also apparent on Fig. 3—in this case due to sample mounting (see Methods). This type of cracking is the subject of the next section.

### Strain-induced cracking (SIC) during uniaxial straining

This part of the study is concerned with applying a longitudinal uniaxial strain to the metallized PDMS samples using the strain applicator. The objective is to observe the effect of adhesion metal type and thickness in the external strain-induced cracking of the metal thin films. As with the PIC study, four types of thin films were evaporated onto PDMS substrates: titanium/gold, nickel/gold, chromium/gold, and gold (see Supplementary Figure S2 for details).

The average crack spacing ${\lambda }_{a}$ and number of cracks (per unit distance) *N* was evaluated to be: gold (*λ*_*a*_ = 47.4 μm, *N* = 0 21.1 mm^−1^), nickel/gold (${\lambda }_{a}=67.1\,\mu m,\,N\,=$ 14.9 mm^−1^), titanium/gold (${\lambda }_{a}=66.7\,\mu m,\,N\,=$ 15 mm^−1^), and chromium/gold (${\lambda }_{a}=136.9\,\mu m,\,N\,=$ 7.3 mm^−1^) at a strain of approximately 5%. Another observation is that the chromium lines resulted in SIC having highly regularly spaced cracking—at least relative to the other films. The SIC in chromium films was also always perpendicular to the line edges—resulting in rectangular mesa structures upon straining.

Chromium was chosen as the adhesion metal for the remaining part of the study. First, few PIC is observed using thin chromium films. Second, chromium/gold is deemed a favourable test combination for the study of SIC. Third, regular sized metal mesas have potential applications^51^.

### Evolution of SIC of chromium/gold thin film on PDMS

**Figure 4:**
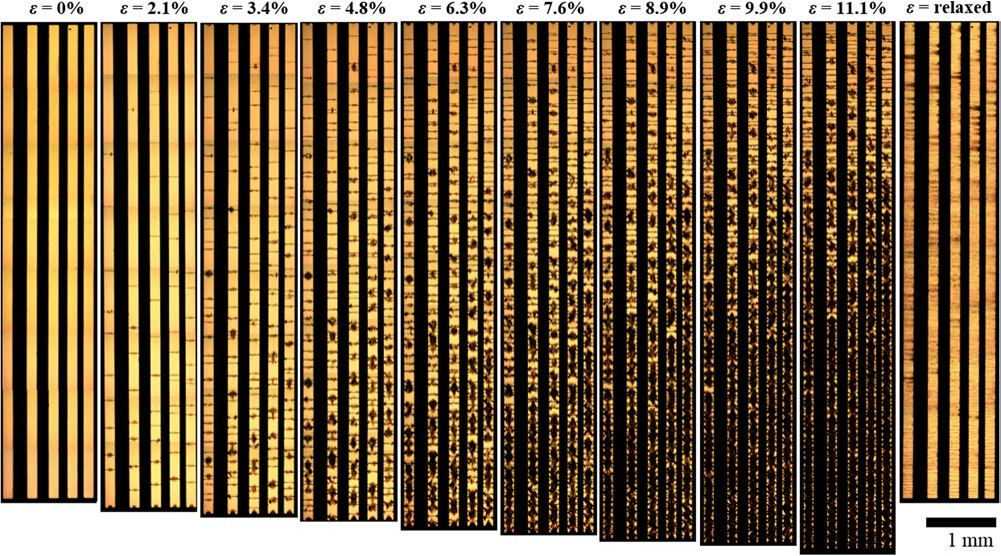
Evolution of strain-induced cracking of thin film, chromium/gold metal lines on PDMS as a function of applied uniaxial strain. From left to right the strain is 0% to ~11%. The image on the far right shows the sample in the ‘relaxed’ state at zero strain after testing. The metallization is thermally evaporated chromium/gold (0.9 nm/21.6 nm) on PDMS. The strain is applied parallel to the direction of the lines.

Figure 4 shows the effect of uniaxial longitudinal straining on chromium/gold (0.9 nm/21.6 nm) metallized lines on PDMS. The thickness of adhesion film is low to ensure no PIC—and less SIC is observed with thin chromium as the adhesion layer.

Several observations are apparent in Fig. 4. First, at zero strain, no PIC is observed in chromium/gold when the chromium thickness is 0.9 nm. Second, the number of SIC increases with applied strain. Third, the mechanical Poisson effect is visible in the lines—resulting in compression of the chromium/gold in the transverse direction leading to buckling which alter the optical reflection of the metal films—this is seen as dark patches on the metal lines of Fig. 4. If we compare the first (zero strain) and penultimate image (11.1% uniaxial strain)—the largest gap between the metal lines reduces from 217 µm to 206 µm—this corresponds to ~5% compression and agrees well with the measured Poisson coefficient of 10:1 PDMS^52^ (see Supplementary Table S2 and Supplementary Figure S3). It is also apparent that the Poisson effect is not uniform along the length of the sample—this is indicated by the dark patch being more apparent at the bottom of the sample. This effect is caused by sample mounting (see Electromechanical Testing). In terms of measuring the overall strain applied to sample—each experiment enabled a calculation of Δ*L*/*L* which was compared to the setting of the testing machine.

### Crack width of chromium/gold thin films on PDMS

**Figure 5:**
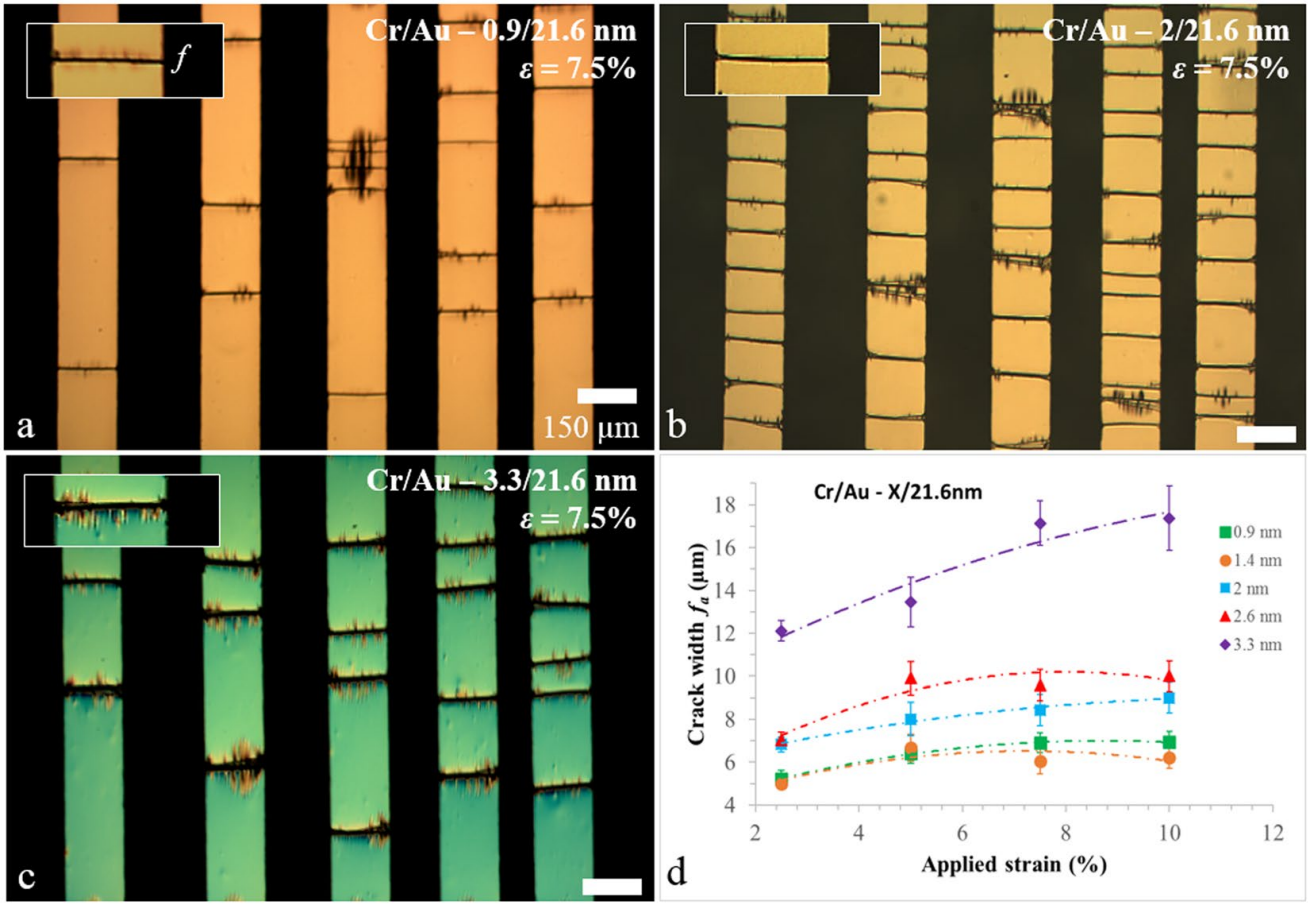
Strain-induced crack width f as a function of chromium metal thickness for metallized PDMS. (**a**) Chromium/gold (0.9/21.6 nm) lines on PDMS. (**b**) Chromium/gold (2/21.6 nm) lines on PDMS, and (**c**) chromium/gold (3.3/21.6 nm) lines on PDMS. The applied strain is 7.5%. (**d**) Plot of the experimentally measured average crack width *f*_a_ as a function of applied strain *ε* and chromium film thickness. The scale bars are 150 µm.

Figure 5 shows the effect of chromium thickness on the perpendicular strain-induced cracking—the figure shows an example of a fixed overall strain of 7.5%. The chromium thickness in Fig. 5 is 0.9 nm (Fig. 5a), 2 nm (Fig. 5b), and 3.3 nm (Fig. 5c). Figure 5d shows a plot of the average measured crack width *f*_a_ (measured using 3D optical profiling—see Supplementary Figure S4) as a function of applied strain *ε* and chromium film thickness.

There are several points to note here. First, uniaxial straining results in perpendicular SIC appearing along the whole length of the chromium/gold lines. Second, a 0.9 nm layer of chromium results in less SIC than a 2 nm thick layer for a given value of strain—we suggest that this observation can be explained since experimental studies have shown that the ultimate tensile strength of thin chromium film is observed to increase with diminishing film thickness^44^. A thicker layer of chromium (3.3 nm) also results in a lower number of SIC compared to 2 nm—in this case the thickness, and the characteristic crack spacing, of the cracking is large, suggesting mechanically robust mesas^45^. In terms of the literature—by evaporating chromium films (thickness range = 50–300 nm) onto polyimide films, Cordill *et al*.^53^ demonstrated that the average crack spacing reduces with reducing film thickness—they also showed that the fracture stress increases with diminishing thickness. Jin *et al*.^54^ observed periodic parallel cracking of chromium films (thickness range = 15–140 nm) on polyethyleneterephthalate (PET) substrates. They observed a higher density cracking for *thinner* films and film buckling due to the Poisson effect—the buckling density was higher for thinner films. Figure 5d shows how the crack width varies with chromium thickness and applied strain. First, increasing the strain results in a larger crack width—this is understandable in terms of a simple spring-type model which takes into account local delamination at crack sites^44^. Second, decreasing the chromium thickness leads to a tendency for the crack width to decrease for a given strain. For flexible substrates the adhesion film thickness plays a major role in defining the cracking process for thin films^55^. It has also been observed using graphene oxide films on PDMS that the width of cracks is thickness dependent: when the thickness varies from 135–300 nm—the crack width varies from 0.28–3.47 µm^45^. In addition, Sakorikar *et al*.^45^ also demonstrated that the average ‘crack density’—related to the reciprocal of the average characteristic crack spacing—diminishes with film thickness, but is also depended on the applied strain ramp rate. For lower ramps rates, the crack density is smaller. The strain ramp rate in our experiments was ~1% s^−1^—we did not investigate the effect of this parameter on cracking in the current study.

### Average crack spacing and crack density in chromium/gold thin films on PDMS

**Figure 6:**
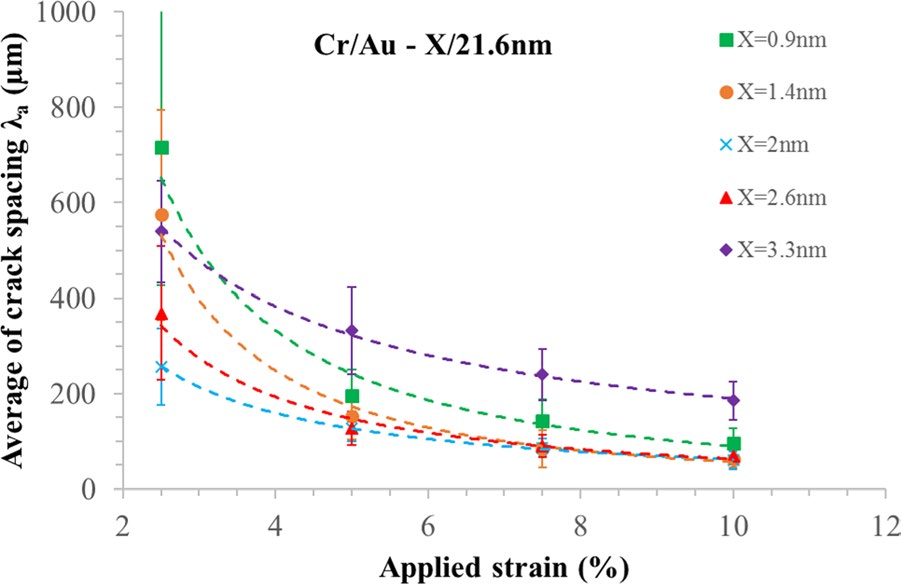
Average of crack spacing as a function of applied strain for chromium/gold lines on PDMS. The chromium film thickness varies from 0.9 nm to 3.3 nm. The gold thickness was 21.6 nm. The strain was applied parallel to the direction of the lines. The data was gathered using several metal lines measuring 7.95 mm by 150 µm. For numerical values, see Supplementary Table S4.

Figure 6 shows the experimental average crack spacing ${\lambda }_{a}$ plotted as a function of applied strain (up to *ε* = 10%) for 5 different chromium film thicknesses ranging from 0.9 nm to 3.3 nm—again the overlaying gold thickness was 21.6 nm. The average crack spacing ${\lambda }_{a}$ and the error (standard deviation—see Supplementary Figure S5, Supplementary Table S3 and Supplementary Figure S6—for a normal probability plot of the data) of ${\lambda }_{a}$ is calculated using between 35 and 133 cracks spacings, depending on the metal thickness and the applied strain.

It is apparent that at low strains (<3%) the average crack spacing for thick chromium (3.3 nm) is lower than for thinner films. This is not what would be expected but can be explained by the fact that at low strains there is PIC in thicker chromium layers—this results in smaller crack spacing. For thinner chromium films there is less PIC—this results in a larger crack spacing. At one applies strain, the SIC dominates the behaviour—at least comparing 3.3 nm to the thinner films. However, a second point to note is that the chromium properties (work of fracture and elastic modulus) are thickness dependent in a non-trivial way.

**Figure 7:**
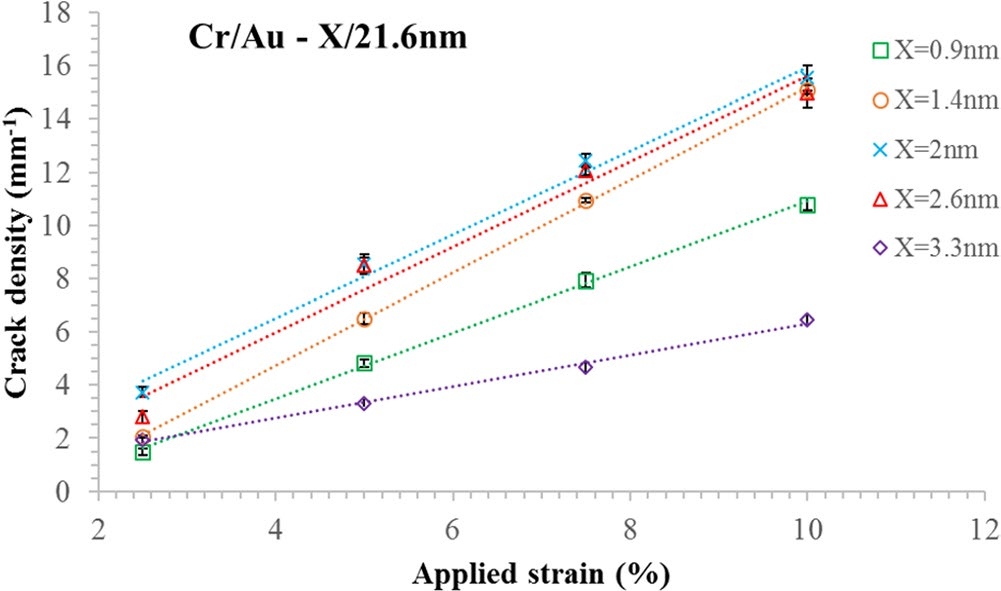
Strain-induced crack density as a function of applied strain. Five different chromium film thicknesses are shown ranging from 0.9 nm to 3.3 nm. The strain was applied parallel to the direction of the lines. The data was gathered using several metal lines measuring 7.95 mm by 150 µm. For numerical values, see Supplementary Figure S4.

Figure 7 shows the strain-induced crack density (number of cracks per millimetre) plotted as a function of applied strain for the five different evaporated chromium film thicknesses ranging from 0.9 nm to 3.3 nm—there is a 21.6 nm gold covering. The average number of cracks (per unit distance) for all chromium thicknesses is seen to vary linearly as a function of applied strain. The numerical values of the experimental data can be found in Supplementary Table S4 of the Supplementary Information. The slopes and values of *R*^2^ can be found in the Supplementary Table S5—*R*^2^ is near unity. It is interesting to note that using a mechanically machined physical ‘shadow’ mask leads to relatively rough line edges—at least compared to those which would have been obtained using lithography. It is well known that in general edge defects^56^ lead to stress concentration which can inevitably result in fracture. The regularity of the crack spacing and number of cracks (per unit distance) is shown by the relatively small standard deviations in Fig. 7. This suggests that the random line side roughness—associated with the physical mask—is *not* a key factor in determining the crack spacing. Indeed, the periodicity of the cracking would be of practical importance if the method were to be applied, for example, to making regular metallic features—e.g. metamaterials on soft matter^51^.

It is interesting to note that the error bars are smaller in Fig. 7 than in Fig. 6. The data suggests that the number of cracks (per unit distance) is constant from line to line. In contrast, the data suggests that the average crack spacing can vary somewhat—whilst still retaining the number of cracks (per unit distance). In this way, Fig. 7 gives a more overall picture of what is going on here whereas Fig. 6 shows more the variations on a local scale.

The regularity of the SIC is reflected in the Gaussian cracking spacing distribution (Supplementary Figure S6). Moreover, one can see that the degree of ordering of the cracking depends on both, the applied strain and the chromium thickness (Supplementary Figure S7).

Following these observations, we can conclude that for a thin adhesion film there is little PIC. By increasing thickness of adhesion film, more PIC appears on the lines until 4.1 nm when PIC becomes wavy (as shown in Fig. 3). Concerning SIC, more and more cracks appear in the lines by increasing the thickness of adhesion film to 2 nm (as shown in Figs 6 and 7). Indeed, after this limit, we observe an increase in the width of the cracks (as shown in Fig. 5) rather than creating more cracks (see Supplementary Figure S8).

### Analytical model for crack spacing of periodic cracks in a thin film

In an effort to understand the experimental observations concerning the cracking of the longitudinally orientated lines one can look to existing models for explanations. It is well known that a thin brittle film—deposited onto, and subsequently supported on a flexible substrate—can fracture into a pattern of parallel cracks^49,50,57–63^. This cracking can be of a spontaneous nature^50,58^—initiated by random process defects^64^, or controlled for practical applications—using deliberately pre-patterned defects^65,66^ or by the presence of boundaries^44^. In the latter cases, the regularity, i.e. ordering, of the cracking is improved.

In terms of modelling these effects, there has been much literature—for example, see the theoretical review of thin film cracking^67^. The characteristic crack spacing *λ* of periodic cracks in a thin film having a thickness ${t}_{f}$ under uni-axial strain has been modelled by the following equation:^49,58^

1 $$\lambda =5.6\sqrt{\frac{{t}_{f}{{\rm{\Gamma }}}_{f}}{{{\varepsilon }_{f}}^{2}{\bar{E}}_{f}}}$$

where ${\varepsilon }_{f}$ is strain of the film $({\varepsilon }_{f}={\rm{\Delta }}{L}_{f}/{L}_{f})$—${L}_{f}$ is the zero-strain length and ${\rm{\Delta }}{L}_{f}$ is the length increase, ${\bar{E}}_{f}={E}_{f}/(1-{\nu }^{2})$—where ${E}_{f}$ is the elastic (Young’s) modulus of the film, $\nu$ is the Poisson coefficient of the film, and ${{\rm{\Gamma }}}_{f}$ corresponds to the *work of fracture* of the film—having units of Jm^−2^. There are some points to understand before proceeding. First, when a metal film is patterned onto a flexible substrate which is subsequently strained using an overall strain $\varepsilon$ (as is the case in our practical study), the strain in the film ${\varepsilon }_{f}\ne \varepsilon .$ The overall strain $\varepsilon$ applied to the system can be linked to the actual strain in the thin film ${\varepsilon }_{f}$ using analytical modelling^44^ which takes into account sample dimensions, the mechanical properties of the materials (and their variation with film thickness) and the number of cracks and dimensions. Second, the Young’s modulus of metal films is known to vary as the film thickness diminishes. Third, the ultimate tensile stress (*uts*) is related to the work of fracture ${{\rm{\Gamma }}}_{f}$ of thin metal films—it is known to vary as the thickness of the film diminishes. Fourth, the uniformity of nanometre and sub-nanometre films—and its influence on mechanical properties—is important^68^. Fifth, thin films are well known to contain residual stress due to processing^48,69^. Such stresses cause PIC (as observed above) and even if no cracking is present a certain residual tensile or compressive stress can be present in the film. For example, in the case of high tensile stress this will lead to film cracking at small applied strain values—effectively shifting the cracking data on the strain axis. Finally, in theory^70^ there will be no mutual interaction between the cracks as their spacing is much larger than the film thickness.

**Figure 8:**
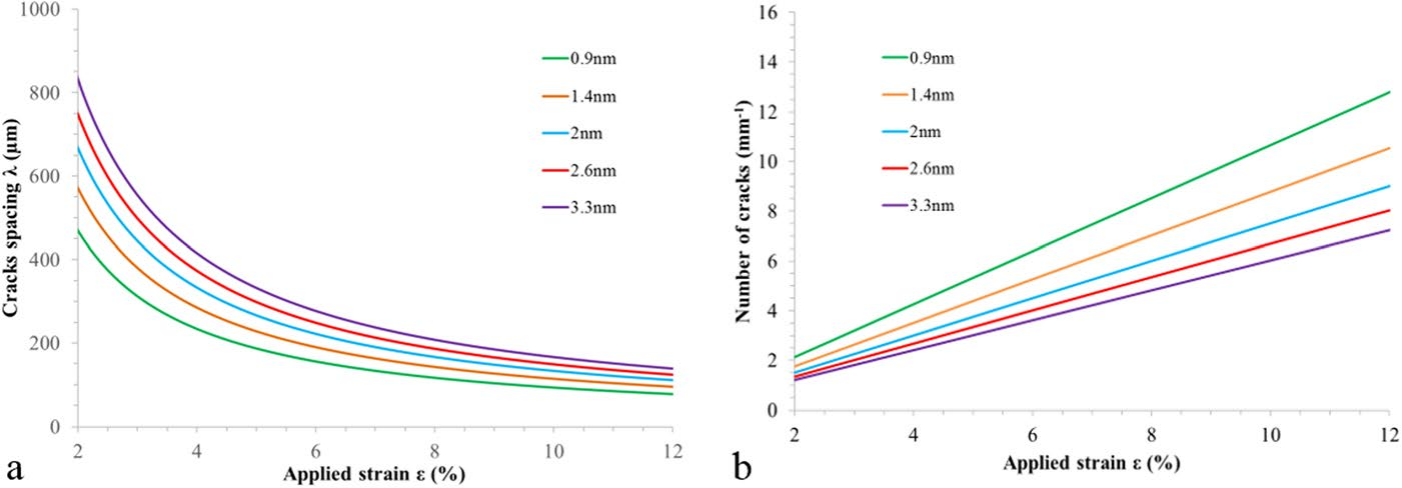
Modelling of strain-induced cracking of a thin brittle film on a flexible substrate. (**a**) Crack spacing as a function of strain. (**b**) Number of cracks (per unit length) as a function of strain.

Based on equation (1), Fig. 8 shows the predicted value of the characteristic crack spacing and the number of cracks (per unit distance) as a function of *overall* strain $\varepsilon$ and chromium thickness—the thicknesses used for the modelling correspond to those used in the experimental section. It should be noted that Fig. 8 takes into account the thickness dependence of the Young’s modulus. In terms of thin evaporated chromium films, based on experimental data in the literature, the Young’s modulus of chromium films can be fitted by an analytical function^44^—${E}_{Cr}=1.66\times {10}^{-12}{{t}_{Cr}}^{0.122}$. In contrast, if the thickness variation of the work of fracture is taken into account, the crack spacing becomes quasi-independent of film thickness (see Supplementary Figure S9)—this is not observed in the experimentation, suggesting perhaps that the *uts* cannot be simply extrapolated to the nanometre and the sub-nanometre domain. In order to explain this the relationship between the overall strain $\varepsilon$ and the film strain ${\varepsilon }_{f}$ is important as it takes into account the strain in the cracks and delamination^44^.

First, consider the general trends of the model and the experimental data. In principle if the mechanical properties of the metal film were independent of thickness we would have $\lambda \propto 1/{\varepsilon }_{f}$ with λ *increasing* as the square root of the thickness at a fixed strain. The number of cracks (per unit distance) would be a linear function of strain—with thicker films giving less number of cracks per unit distance. All the results plotted in Fig. 8 agree very well with this linear dependence—even up to relatively large strain values. However, the predicted trend of the thickness is not apparent. In an effort to explain this, we can question how the ratio ${{\rm{\Gamma }}}_{f}/{\bar{E}}_{f}$ varies with film thickness. The bulk mechanical properties of the materials used in the current study are relatively well known. In contrast, the mechanical properties (residual stress, ultimate tensile strength, elastic modulus…) of thin films are much less known—especially for nanometre and sub-nanometre film thickness—and depend on deposition method, process parameters, film thickness, and host target substrate. For a specific thickness, the trends of Fig. 8 correspond well with the experimentally obtained Figs 6 and 7: the crack spacing decreasing with increasing strain—this is clearer on the plot of number of cracks (per unit distance) which is linear.

Using the experimental data obtained from Supplementary Figure S2 and Fig. 7 it is possible to extract values for the fracture toughness ${K}_{Ic}=\sqrt{{{\rm{\Gamma }}}_{f}{E}_{f}}$—these values are given in Supplementary Table S6. Fracture toughness is an important parameter in materials science^71^, it describes the ability of a material to resist fracture—an important property to consider for many modern applications^72^.

The fracture toughness of bulk gold is in the range 40–70 MPa $\sqrt{m}$ whereas the fracture toughness of bulk nickel is in the range 100–150 MPa $\sqrt{m}$—the same order of magnitude as the fracture toughness of titanium and its alloys. For chromium, the published bulk value of the fracture toughness is higher ~400 MPa $\sqrt{m}\,$^73^. In terms of thin films, facture toughness is known to reduce as a function of film thickness^68,74–77^. First, the value of the fracture toughness of the ~20 nm thick gold film (deposited onto PDMS) here is much less than the bulk value and an order of magnitude smaller than that published in the literature for thin films whose thickness ranges from 60–320 nm^68^. The values of the fracture toughness of nickel, titanium, and chromium are also much lower than their corresponding bulk values. Interestingly, the fracture toughness of the chromium films varies a little with thickness—although this is a small range 0.9–3.3 nm. The mechanical properties of uniform chromium films can vary greatly from the bulk in the nanometre thickness range. For example the Young’s modulus varies from 180–279 GPa, and the critical failure strain varies from 1.9% down to 0.16% in the thickness range 15–500 nm—for the thickness range here is 0.9–3.3 nm, their mechanical properties are not documented. Second, the impact on the mechanical properties of the *uniformity* of the chromium film at nanometre and sub-nanometre thicknesses when deposited onto flexible supports also remains undocumented. On this last point, the AFM measurements indicate a uniform chromium film over the thicknesses deposited. Finally, the process induced residual tensile stress in thin chromium films is thickness dependent^78^ and known to be generally high ~1–2 GPa^48,69^.—although, again, less is documented concerning sub-nanometre chromium films. On this last point, residual tensile stress in the film would—in principle—reduce the observed value of fracture toughness. Finally, the measurements indicate that the relationship between cracking of a thin nanometre metal on an elastomer substrate film by imposing an external strain is not trivial at such thin film thicknesses where the ratio ${{\rm{\Gamma }}}_{f}/{\bar{E}}_{f}$ is certainly thickness dependent.

To conclude the first part of the paper, several points can be noted. First, chromium is the more suitable metal for the study of long lines on PDMS. Second, the thickness of the adhesion layer plays a critical role in the cracking. To obtain a low number of cracks and thin cracks under strain, a very thin adhesion film is required. Despite these parameters, the electrical characterization remains open circuit for longitudinally-strained on longitudinally-orientated metal lines—even at small strains. Hence, for mechanically and electrically robust lines, we reorient the sample by 90°—taking advantage of Poisson effect—and applied longitudinal strain to transversally-orientated metal lines. Few studies have investigated electrical characterization at high strain on long metal lines orientated in this way.

### Thin-film metal lines transversally-orientated to the uniaxial longitudinal straining of the sample

For the remainder of the paper all the samples are mechanically strained having the longest length of the metal lines orientated *transversally* to the direction of the applied strain. In this case the mechanical Poisson effect causes the longest length *L* of the lines (and also the film thickness *t* to be in compression—whereas the width *w* of the lines is in mechanical tension. Under such conditions, we can apply longitudinal strain to the PDMS sample and measure the evolution of the line’s electrical resistance (piezoresistance) along the length *L* of the wire—transversally to the direction of the applied strain. In order to measure the electrical resistance of the lines and to observe the combined effects of cracking and the Poisson effect, two types of probe tips were used (see Methods and Supplementary Figure S10). The electrical resistance was first measured using probes whose tip radius was much smaller than the width of the line. Following this, the electrical resistance of the lines was measured with probes which ensured a contact along the whole width of the line—at the ends of the line. In order to perform the measurements, the lines were separately strained, imaged using various methods, and then probed electrically.

**Figure 9:**
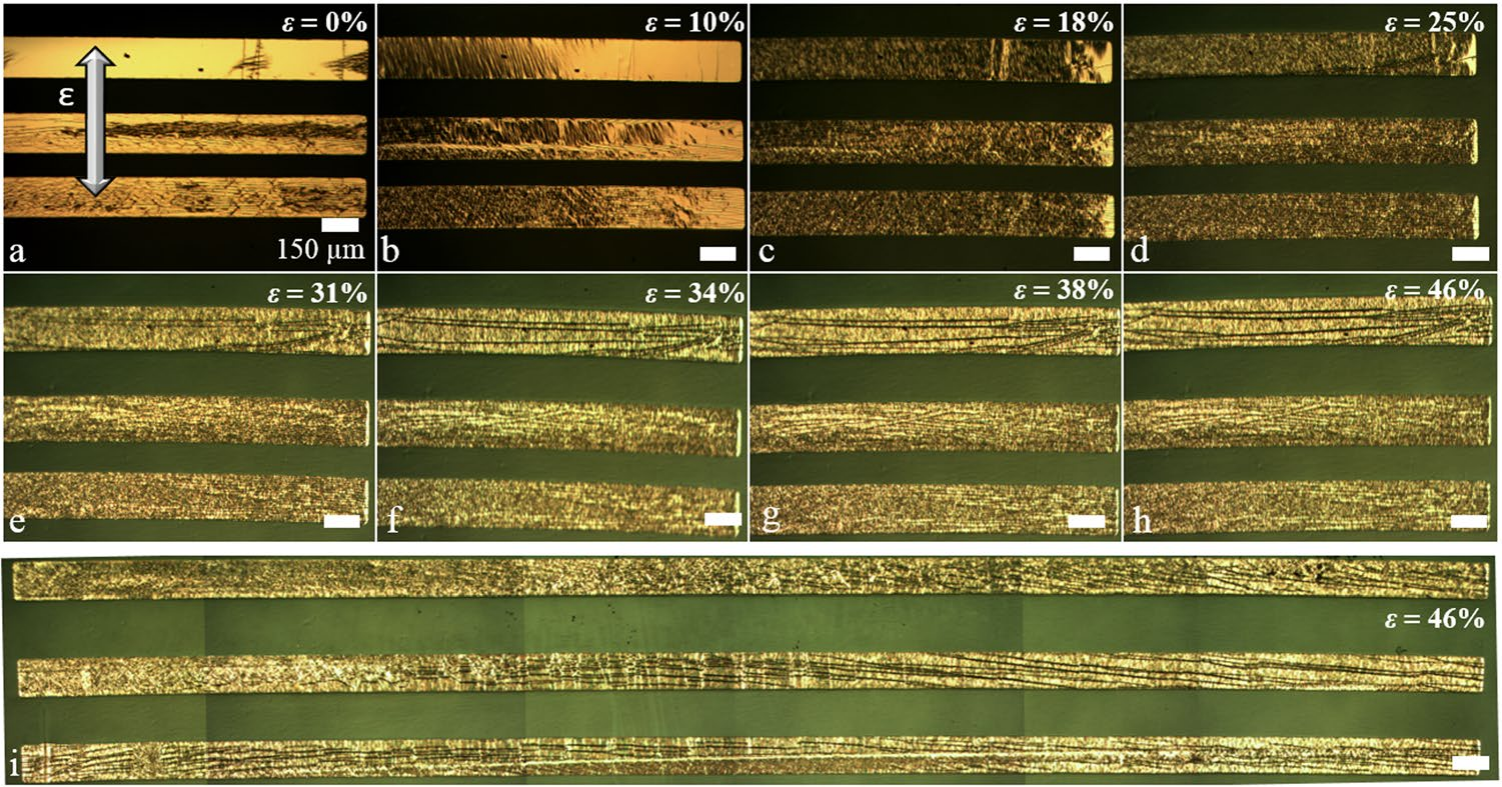
Optical microscope images showing longitudinally-strained, transversally-orientated thin film chromium/gold (0.9/21.6 nm) lines evaporated onto PDMS. (**a**) to (**h**) The strain varies from 0% to 46%, the length of the lines is 2.62 mm and the width is 150 µm. (i) A long chromium/gold metal line under 46% strain. The length of the line is 7.95 mm. The scale bars are 150 µm. The arrow indicates the direction of the longitudinal straining of the sample—the metal lines are transversally orientated with respect to this straining.

Figure 9 shows optical microscope images of the chromium/gold lines under strain testing. Two lengths of lines were used for this part of the study—2.62 mm and 7.95 mm. Here the chromium/gold thickness was chosen to be 0.9/21.6 nm as this thickness avoids PIC and results in little SIC, this is important for electrical characterization.

This configuration of transversally-orientated lines revealed some interesting mechanical behaviour. First, as the strain is increased the Poisson effect becomes visible in the images—this is due to film buckling (see Fig. 9a–h). Second, cracking appears in the lines as the strain is increased—but the cracking is now parallel to the longest length of the thin film metal lines—see Fig. 9a–h (and Supplementary Figure S11 for 2D and 3D profile cracking). This was also visible in very long lines—see Fig. 9i. Finally, some of the longer lines contained PIC orientated perpendicularly to the long length of the lines—microscopy revealed that the crack widths of the PIC diminished upon increasing the strain, presumably due to the Poisson effect. Let us now look at the electrical results of these various thin film metal lines on PDMS strained in the transverse configuration.

### Results using small tip probes

**Figure 10:**
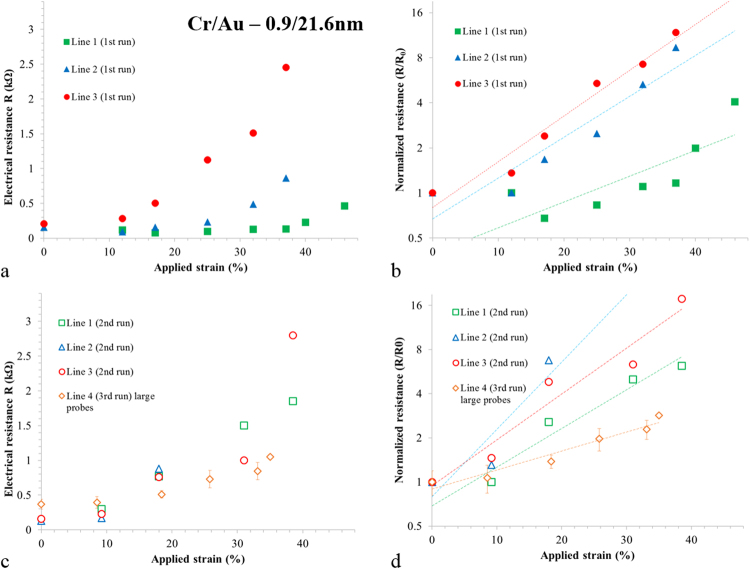
Summary of the electromechanical characterization. (**a**) The measured electrical resistance of the first strain sweep plotted as a function of strain using small tipped probes. (**b**) The normalized electrical resistance of the first strain sweep using small tipped probes. (**c**) The electrical resistance of the subsequent sweeps using small and large tipped probes. (**d**) The normalized electrical resistance of the subsequent sweeps using small and large tipped probes.

Figure 10 shows a summary of the electrical results obtained when testing the thin film lines (*L* = 2.6 mm). First, all current-voltage traces were linear indicating ohmic behaviour (see Supplementary Figure S12). Second, the slope of the *IV* curves was dependent on the applied strain. In general, the transversally-orientated thin film lines conducted at zero-strain—indicating no PIC—and up to 37% longitudinally applied strain. The electrical resistance of the lines was seen to increase non-linearly with applied strain (see Fig. 10a) this is well-known for metalized PDMS strained longitudinally along the longest lengths of the lines, where micro-cracking contributes to an increases in the electrical resistance^17^. The increase of transversally-measured electrical resistance with strain is apparent in Fig. 10a which shows the resistance of 3 lines plotted as a function of applied strain—it should be noted that these results are for the first strain sweep using previously unstrained samples. Using the small tipped probes two types of behaviour were observed—these are best seen when the *normalized* resistance (*R*/*R*_0_) (see Fig. 10b) is logarithmically plotted as a function of applied strain. For most lines the transverse electrical resistance increases rapidly with strain^19,79^. This plot reveals that there are two general types of behaviour for lines—a rapid increase of the resistance (filled red and blue symbols) with strain and a less rapid increase (filled green symbols). When the lines are tested again electrically using a second and third strain sweep their electrical behaviour is observed to have been modified. Now the resistance increases rapidly with strain (open data points)—see Fig. 10c. Again, this is best seen if the normalized resistance is plotted against strain—see Fig. 10d. The main observation here is that a stable electromechanical behaviour is achieved for all lines *after an initial strain sweep*—presumably due to the influence of the cracking.

**Figure 11:**
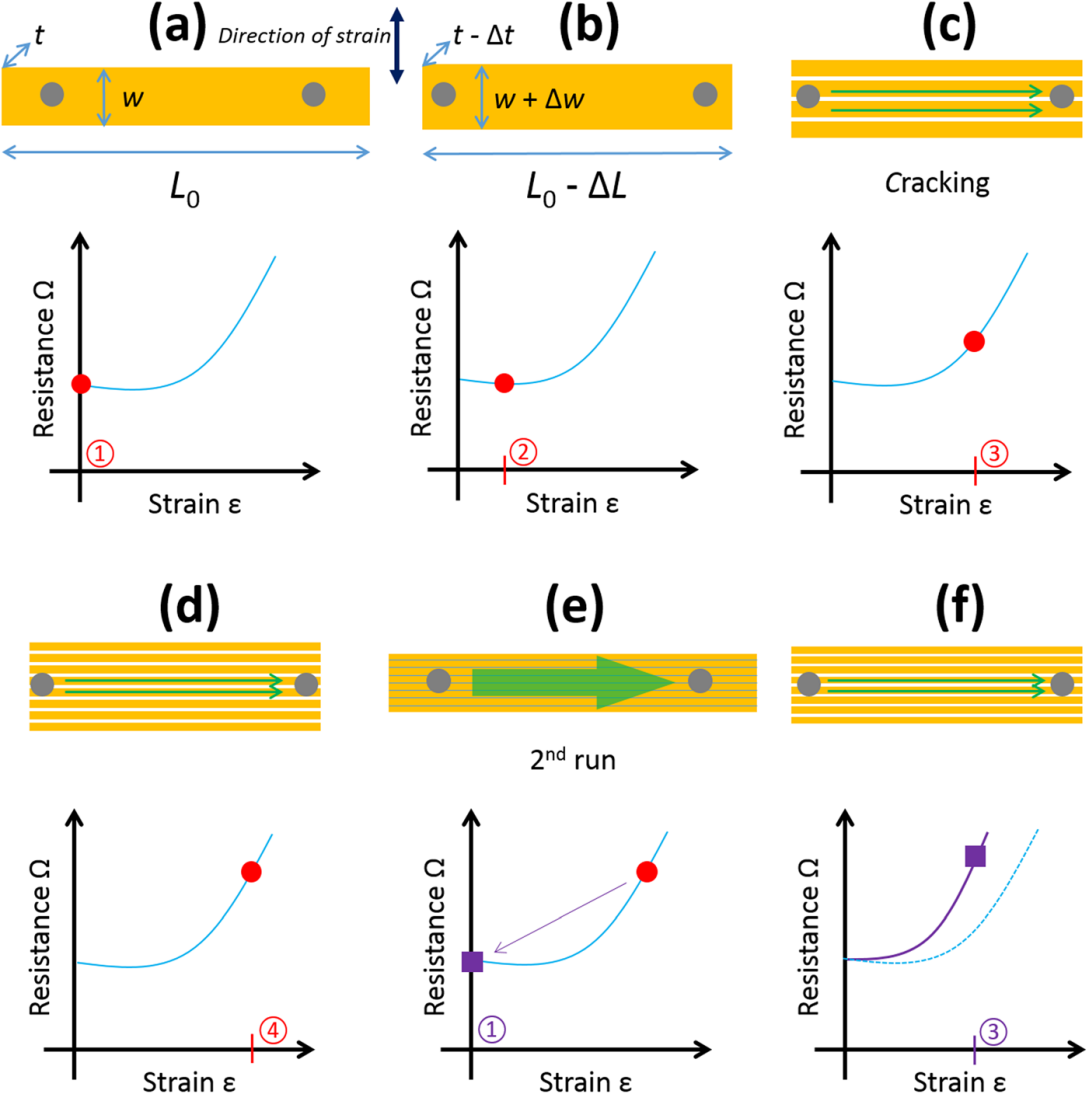
Probing lines transversally-orientated to the strain using small tipped probes. (**a**) Zero-strain condition ① and electrical resistance. (**b**) Small strain condition ② before initial cracking. (**c**) Initial cracking leads to the appearance of long transversal cracking at higher strain condition ③. (**d**) High strain condition ④ leads to more cracking. (**e**) Zero-strain relaxed condition ① following the first strain-sweep and (**f**) n^th^ sweep strain condition ③ as in (c). The circled numbers refer to specific values of strain. The two probes contact areas are indicated by grey circles.

In an effort to understand these observations obtained using the small tipped probes, Fig. 11 shows a schematic diagram of the effect hypothesized ideal parallel cracking—along the length *L* of the lines—on the electrical resistance of the lines measured between two small contact surface probes indicated by the grey circles.

**Figure 12:**
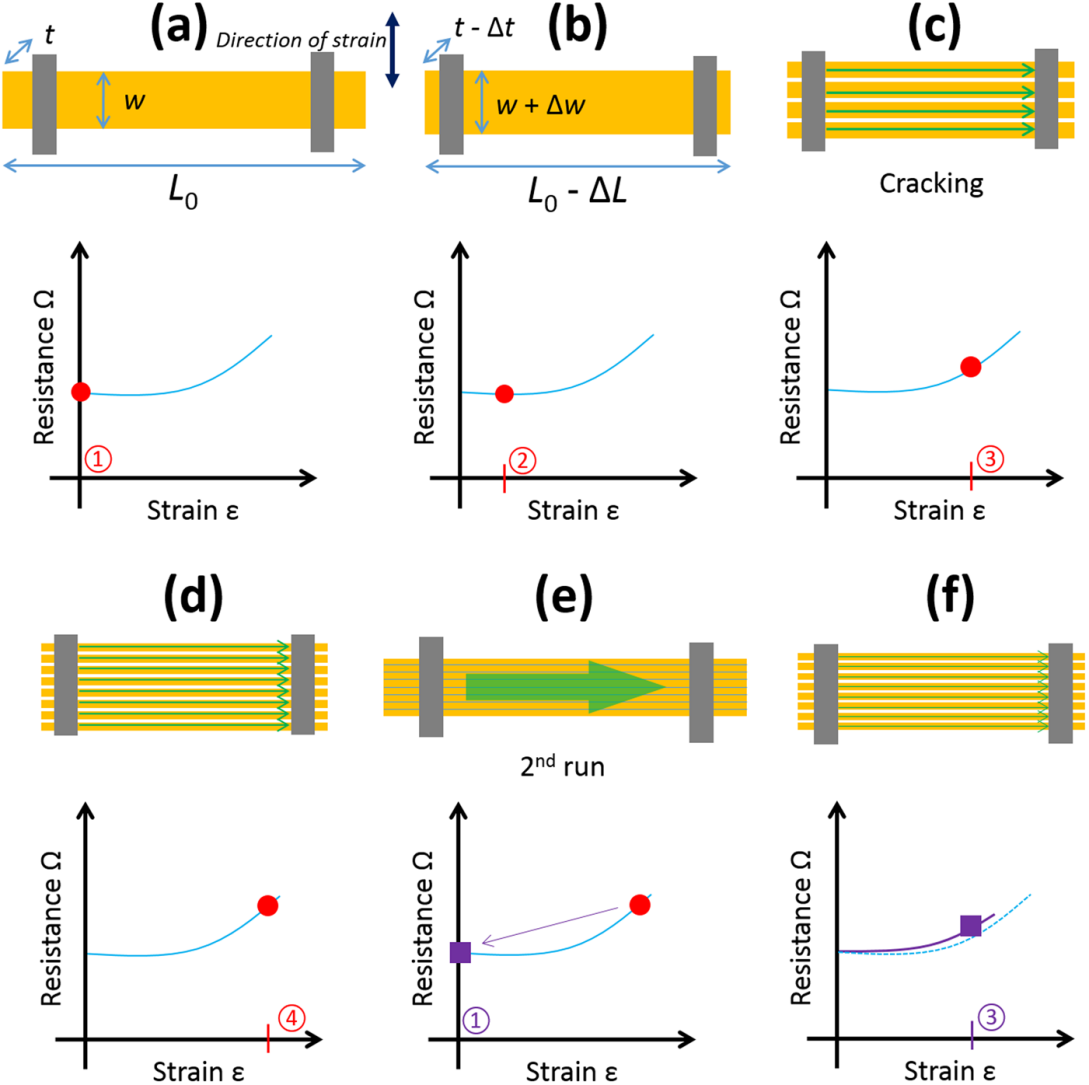
Probing lines transversally-orientated to the strain using large tipped probes. (**a**) Zero-strain condition ① and electrical resistance. (**b**) Small-strain conditions ② before initial cracking. (**c**) Initial-cracking leads to the appearance of long transversal cracking at higher strain condition ③. (**d**) High strain condition ④ leads to more cracking. (**e**) Zero-strain condition ① following the first strain-sweep and (**f**) *n*^th^ sweep strain condition ③ as in (c). The circled numbers refer to specific values of strain.

If we assume that there is no PIC and no residual stress in the film, then the line has a zero-strain resistance defined purely by its geometry (*w*, *L*, and *t*) and electrical resistivity—see Fig. 11a. If a small strain (circled red 2) is applied longitudinally to the sample, the line length *L* (and its thickness *t*) will be in compression—see Fig. 12b. In this case the electrical resistance of the lines falls a little due to geometrical changes—and ignoring energy band related effects in metals^80^. Upon increasing the longitudinal strain further (circled red 3), the first horizontal cracking appears—as the width of the line *w* is in tension—see Fig. 11c. As the probe tips are smaller than the line width *w*, only some of the horizontal paths will be available to conduct electricity—this results in an increase of the measured electrical resistance. Increasing the longitudinal strain further (circled red 4) leads to more horizontal cracking—leading to a further increase in the electrical resistance—see Fig. 11d. When the externally imposed longitudinal strain is reduced to zero—see Fig. 11e, the cracked lines are again physically contacted—the result of this is a zero-strain resistance equal to the uncracked zero-strain resistance initially observed—*c.f*. Figure 11a. Interestingly, when the same longitudinal strain (circled purple 3) is applied (as in Fig. 11c)—as we now have more horizontal cracking (since we have previously applied a larger strain (as in Fig. 11d)—the measured electrical resistance is higher than that which was measured at the same strain during the first cycle. The initial strain sweep induces cracking which leads to stable electromechanical behaviour in subsequent strain sweeps. This is what is shown when comparing the filled data points (initial strain sweep) and the open data points (second strain sweep) in Fig. 10.

### Results using large tip probes

The transversally-orientated lines were tested electrically using large tips which ensured a physical contact with the whole width of the line. It was again observed that the longitudinal strain applied to the sample appeared between the lines i.e. the distance between the lines increased. As above, long horizontal cracks appeared in the lines. The electrical resistance of the lines is plotted as a function of applied longitudinal strain to the sample—indicated by the open gold diamond in Fig. 10c. The normalized resistance is plotted logarithmically in Fig. 10d—open gold diamonds explications. It was observed that the electrical resistance of the lines increased from ~350 Ω to ~1000 Ω from 0% to 46% applied strain—despite the formation of several long horizontal cracks. In terms of the zero-strain resistance expected for the lines, taking the resistivity of gold and the chromium to be 22 nΩ m and 125 nΩ m respectively, one can expect a zero-strain resistance of ~17 Ω for the metal lines tested here. The average measured zero-strain resistance of all lines is 160 ± 47 Ω before the application of strain and 196 ± 91 Ω after the application of strain—indicating that micro-cracking^19,37^, which leads to a discontinuous metal film^81^, is determining the zero-strain resistance.

Again, in an effort to understand the observations Fig. 12 shows a proposed model for how system is evolving with applied longitudinal strain.

**Figure 13:**
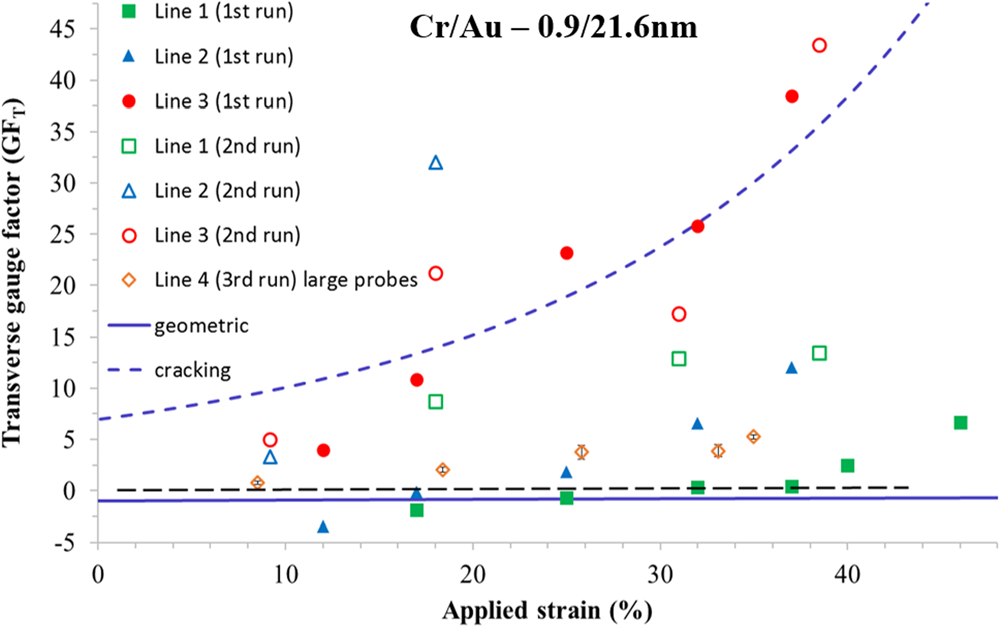
The transversal gauge factor(*GF*_*T*_) of the lines plotted as a function of applied strain. The filled data points correspond to the first strain sweep on a new sample. The open data points correspond to the second sweep on the same sample. The solid line corresponds to the variation of the gauge factor if purely geometrical effects are taken into account (see Supplementary Figure S13). The long dashed line is the calculated gauge factor based on an average of the experimental data. The short dashed line corresponds to $G{F}_{T}$ = 0.

As above, the zero-strain resistance is some fixed value—see Fig. 12a. The application of a small strain (circled red 2) which causes no cracking leads to a geometrical change resulting in a small reduction of the electrical resistance of the lines—Fig. 12b. Further increasing the applied strain (circled red 3) leads to the formation of ‘horizontal’ cracking in the lines—Fig. 12c—as above in Fig. 12. However, as the probe tip is much larger than the width of the lines, the measured resistance is all *n* lines in parallel. Increasing further the longitudinal strain (circled red 4) leads to more cracking—but again as the probe contacts all the parallel lines the resistance should—at least in principle—remain relatively constant—Fig. 12d. At zero-strain, the electrical resistance returns to the same value of the zero-strain resistance as: (i) all transversal cracking short circuits (ii) the probe size is larger than *w*. Cycling the strain leads to the same resistance values for given strains (circled purple 3)—as there is no electromechanical hysteresis in the system—*c.f*. Figure 13.

### Piezoresistive gauge factor of the transversally-orientated metal lines on PDMS

The electrical results enable the *transverse* piezoresistive gauge factor ($G{F}_{T}$) of the thin film metal lines to be extracted—this can be thought of as analogous to the ${\pi }_{12}$ transverse piezoresistance coefficient in a typical semiconductor piezoresistance setup^82,83^. The value of $G{F}_{T}$ is calculated using the following equation:

2 $$G{F}_{T}=\frac{{\rm{\Delta }}{R}_{T}}{{R}_{T0}}/{{\epsilon }}_{L}$$

where ${R}_{T0}$ is the zero-strain electrical resistance of the line—oriented transversally to the applied longitudinal strain, the change in electrical resistance ${\rm{\Delta }}{R}_{T}$ due to strain is given by ${\rm{\Delta }}{R}_{T}={R}_{T}({\epsilon })-{R}_{T0}$, and ${{\epsilon }}_{L}$ is the *longitudinal* strain applied to *the whole of the sample*. Based on equation (2), the values of $G{F}_{T}$ of all lines are plotted as a function of applied longitudinal strain and shown in Fig. 13.

There are a number of interesting observations to be made here. First, lines tested using the small tipped probes have initially relatively small gauge factors during the first strain sweep—solid data points in Fig. 13. Second, using the large tipped probes, the lines also demonstrate a relatively low gauge factor—open gold diamonds in Fig. 13—and remaining relatively low after an initial strain sweep. At low strains, some of the gauge factors are negative—as would be expected from a purely geometrical effect (see Supplementary Information Section 12)—although this could, at least in part, have a percolation origin^84,85^. However, one must remember that the suggested percolation effects^84^ apply to micro-cracking^37^ in gold on PDMS—whereas here the cracking is much larger having a macroscopic origin in the chromium/gold and along the lengths of the lines. Interestingly, when the lines are re-tested using the small probes, the observed gauge factors are in general higher (open symbols in Fig. 13)—being upwards of 10 and a highest value of ~40 being observed at a longitudinal strain of ~40%—this corresponds to a boost in the gauge factor of metal films of about 60. These values are much greater than what would be expected for a metal film undergoing a purely geometrical change—for example, the solid purple line in Fig. 13 shows the calculated transversal gauge factor (see Supplementary Information Section 12 for derivation) based on a geometrical change of line dimensions *L*, *w*, and *t*. In a purely geometrical case the gauge factor depends only on the applied longitudinal strain: $G{F}_{T}=-\,1/(1+{\varepsilon }_{f})$. The initially cracked lines (filled red circles), on the other hand, have a large *positive GF* which would not be expected for a thin metal line in compression.

Finally, when all lines are re-tested, the initially relatively small negative gauge factors of the lines having little cracking become large and positive—suggesting the cracking and pre-cracking is playing a major role in the piezoresistive behaviour of such thin film on flexible substrates. We suggest that the large $G{F}_{T}$ are predominantly caused by horizontal cracking along the lines longest length—percolation of micro-cracking due to the Poisson effect^84^, as has been suggested to explain some thin film behaviour on flexible supports, leads to a more stable electrical resistance as a function of applied strain.

In terms of metal films, the piezoresistive behaviour depends on whether the film is continuous or discontinuous^81^. In the former case, the geometric effect will dominate and the gauge factor will naturally be low^86,87^. In the latter case, a discontinuous metal film can be evaporated onto a rigid support using a very thin film—this results in an enhanced gauge factor^86,88,89^. A discontinuous film can also be formed on a flexible support via various forms of film cracking^90–94^, both process and strain-induced—this also results in a higher gauge factor. The observed increase in the gauge factor is due to conduction mechanisms in the discontinuous films—resulting from the presence of e.g. metallic islands, metallic nanowires, and various scales cracking. Our results can be compared with other findings—see Table 1.

**Table 1:** Comparison of observed gauge factors to those found in the literature. Values are given for continuous and discontinuous metal films deposited onto rigid and flexible support material.

Thin film	Support/substrate	Direction	GF	strain range	Mechanism	Ref.
Au (>100 nm)	Steel and Al-alloy	L	1–4	<1%	Geometric	Parker and Krinsky^86^
Au (<100 nm)	Steel and Al-alloy	L	10–100	<1%	Non-uniform film	Parker and Krinsky^86^
Au (10–100 nm)	Fused silica	L	2	Not given	Geometric	Reale^87^
Au (3–15 nm)	Glass	L	20–60	Not given	Non-uniform film	Hok *et al*.^88^
Au (50 nm)	Silicon	L	460	not given	Inhomogenized film	Mohanasundaram *et al*.^89^
Cu (188 nm)	Mylar	L/T	2.2/−0.6	<0.1%	Geometric	Rajanna and Mohan^90^
Au (200 nm)	PDMS/Kapton	L	40–75	0–0.6%	None suggested	Wen *et al*.^91^
Au (nanowires)	paper/PDMS	L	7.4	0–12.5%	Cross conduction	Gong *et al*.^92^
Au (200 nm)	PDMS	L	1000	0–0.12%	Micro-cracking	Liu *et al*.^93^
Pt (20 nm)	PUA	L	2000	0–2%	Nano-scale cracking	Kang *et al*.^94^
Au/Cr (20/1 nm)	PDMS	T	5–40	0–40%	Large scale cracking	this work

*L = longitudinal, T = transversal.

Finally, our results can also be compared to the giant piezoresistance effects seen in silicon and its nanostructures^80^. Interestingly, the relatively large *boost* of the gauge factor (~60) at ~40% strain—this is comparable to the boost of the piezoresistive coefficient observed in silicon nanowires^80,95^ where a gain of ~30 times the silicon bulk values of the piezoresistance coefficient was observed.

### Electromechanical ‘self-healing’ of thin film metal lines on PDMS

**Figure 14:**
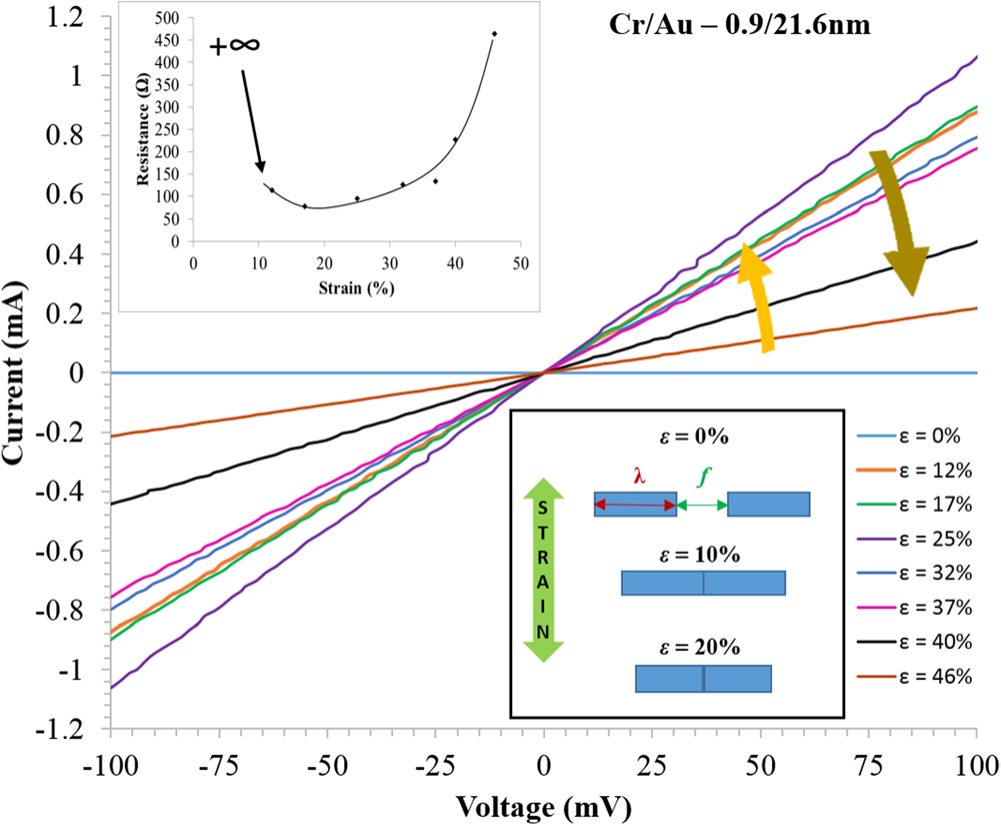
Electromechanical results showing a ‘self-healing’ effect. A plot of the current-voltage curves of chromium/gold (0.9/21.6 nm) thin film lines as they vary with applied strain. The upper inset shows the variation of electrical resistance as a function of strain—initially open circuit at zero-strain. The lower inset shows a schematic diagram illustrating the Poisson effect-assisted short circuiting of the cracks which leads to self-healing.

Finally, it was observed that lines that contained PIC *perpendicular* to the longest line length *L* had an initial zero-strain resistance of infinity—i.e. an open circuit due to cracking. For such lines, as the longitudinal strain is increased some lines began conducting electricity again—this can be seen in the current-voltage curves shown in Fig. 14.

We observe that the mechanical Poisson effect—which leads to the lines being in compression—causes short circuiting of the PIC. Thus, we observe a Poisson effect-induced electromechanical self-healing in the transversally orientated lines. This result can be put in a wider context—as self-healing of thin film technologies is becoming an area of interest^96,97^. The results can be compared to the suggest Poisson effect-related percolation effects observed in metallized PDMS structures subjected to a certain level of strain cycling^84^. Interestingly, it has recently been shown that the Poisson effect can be tailored for applications^98^—suggesting that it could have great importance in flexible applications.

## Conclusions

In conclusion, polydimethylsiloxane (PDMS) can easily be metallized using thermal evaporation via physical shadow masks to form gold lines. The adherence of such lines to the PDMS was enhanced using thin films of titanium, nickel and chromium. Two types of cracking are observed in such systems—process-induced cracking (PIC) and strain-induced cracking (SIC). The former occurs spontaneously due to process-induced residual stresses whilst the latter is caused by straining the samples using a strain applicator. Very little PIC is observed when using evaporated nickel and titanium as an adhesion film for gold. PIC can be virtually eliminated when using chromium as the adhesion layer by reducing the chromium thickness to <1 nm. SIC is observed for the thin film adhesion layers titanium and nickel—at relatively low values of external strain ~5%. In chromium films regular SIC is seen to produce similar sized rectangular mesa structures along the line. The ordering of the resulting metal mesas depends on film thickness and external strain level. The SIC periodic cracking agrees well with analytical models given in the literature. The work of fracture of thin films can be estimated from the approach. When lines are oriented transversally to the applied strain, two interesting behaviours manifest. Firstly, the *transversal* gauge factor $G{F}_{T}$ of the metallized lines can be extracted—transversal cracking in such lines leads to a high value of $G{F}_{T}$ compared to standard thin film strain gauges. We conclude that such an approach may be useful for enhanced sensitivity metal film strain gauge technology working up to large strain values. Secondly, an electromechanical ‘self-healing’ effect is observed in some metallized lines. The mechanical Poisson effect leads to short circuiting of PIC at a critical strain value—leading to the whole line becoming conducting again, up to very high strains ~40%. Finally, metallized PDMS is shown to be a simple-to-fabricate, useful vehicle for fundamental experimental cracking studies which allow models to be put to the test. Cracking in such films allows the observation of original electromechanical behaviour which—if harnessed correctly—could be technologically useful.

## Methods

### PDMS sample preparation

All fabrication processes and measurements were performed in an ISO 5/7 cleanroom. PDMS samples were prepared using a commercially available kit (Sylgard 184, Dow Corning). The base and curing agent were mixed to a ratio of 10:1, degassed using a rotary pump and moulded using a flat-based Teflon dish. The volume was calculated to achieve a thickness of 1.7 mm. The mixture was then cured in an oven at 100 °C for 120 minutes—no extra high-temperature anneal was performance on any samples. At a 10:1 volume ratio, the resulting PDMS has previously been measured to have an elastic modulus of 2.4 MPa^52^. PDMS samples were then cut—using a scalpel—from the main block to have dimensions of 2 mm × 4 mm × 1.7 mm. Care was taken to avoid contact with the PDMS surface. Indeed, even in a cleanroom environment the insulating nature of PDMS leads it to attract pollution e.g. particles—these can be removed using an ultrasonic bath. Just prior to metallization using a physical shadow mask, the PDMS samples were exposed to oxygen plasma. The oxygen plasma power (750 J) and time (10 s) using a Plasmalab 80+ (Oxford Instruments, UK) has already been optimized by the authors to maximize metallization adhesion—but not cause surface cracking of the PDMS^44^.

### Metallization using evaporation via a physical shadow mask

The oxygen plasma-treated PDMS samples were metallized using evaporation—via a physical shadow mask to define lines—in a commercial MEB 550S electron beam evaporation system (Plassys, France) at <10^−7^ mbar. Thin metal films—titanium, nickel, chromium, and gold—were deposited of varying thickness explained in the manuscript. Thin film metallic lines were formed on the surface of the PDMS samples using a shadow mask. This masking method was chosen over lithography for its ease. The physical shadow mask is 50 µm thick and contains lines having dimensions of 100 µm × 500 µm lines (40 features) and 7.95 mm × 150 µm lines (15 features). During evaporation, the PDMS samples were maintained at room temperature using a cooling system. The deposition rates were: 0.2 nm s^−1^ for the titanium, the nickel and the chromium, and 0.5 nm s^−1^ for the gold. Post-evaporation handling can lead to unwanted external strain-induced cracking so care was taking during post-process sample handling to avoid this. The metal shadow masks were carefully removed from the surface of the PDMS after removing the samples from the evaporator. The thin film thickness and roughness values were carefully measured and analysed^99^ using atomic force microscopy (AFM)—using a Dimension D3100 (Bruker–Veeco, USA). The films were evaporated onto polished silicon wafer calibration samples which contained a masked feature to produce a step. The scanning electron microscopy (SEM) imaging was performed using an Ultra-55 (Zeiss, Germany).

### Electromechanical testing

A basic strain applicator^22^ capable of imposing a longitudinal mechanical strain was adapted for the electromechanical testing of the metallized PDMS samples. The PDMS samples were carefully mounted onto the tool two at a time—again taking care to avoid accidental application of strain to the samples prior to testing. For example, the mere action of mechanically fixing the samples sufficiently can induce deformation (bending and swelling due to the Poisson effect) at the edges of the sample. The initial bridge length needed to be fixed with respect to the thin film metal line lengths to avoid this potential problem. There is also a trade-off between the force necessary to avoid sample slipping during application of strain and the edge deformation cause by squeezing the PDMS hold points (approximately 0.5 cm by 1.5 cm each side) too much. When the samples are mounted, the PDMS is squeezed between two metal plates. This causes the free-standing PDMS to swell resulting in a small compressive stress offset. It is observed that this compressive stress offset depends on the PDMS mounting. Squeezing the PDMS sample more at one end than the other can lead to the effect seen in Fig. 4a slight uneven Poisson effect along the sample. Once the samples are in place they can be observed optically (Leica, Germany) and tested electrically using a 2612 System SourceMeter® (Keithley, USA) and driven using LabView® software. Two types of metallic probes were used for the two-terminal testing. The first were tungsten PTG20-10 commercial probes (Microworld, France) which have a 10 µm tip radius. The second were the above probes which had been mechanically modified to have a tip radius of ~750 µm. In addition, the topography of the samples was studies using interference microscopy with a Contour GT-X 3D optical profiler (Bruker Corp., USA). The strain tool allows a stress resolution of 0.1% when *L*_o_ of the PDMS is 1 cm. The measurement involved increasing the external strain in steps (ramp rate ~1% s^−1^), recording optical photographs of the film and subsequent cracking (if there is any) followed by electrical testing if a continuous film (no cracking) is observed. Following maximum external strain, the sample is returned to zero-strain—in this state care is taken to observe any PDMS buckling which would indicate sample slipping during the measurements. In the absence of buckling, photographs are taken of the final zero-strain state of the metallization.

### Data availability

All data generated or analysed during this study are included in this published article (and its Supplementary Information files).

## Electronic supplementary material

Supplementary Information
