# Supplementary material for: Cracking effects in squashable and stretchable thin metal films on PDMS for flexible microsystems and electronics
Source: Sci Rep. 2018 Jun 22;8:9492. doi: 10.1038/s41598-018-27798-z (PMC6015027; doi:10.1038/s41598-018-27798-z)
Supplement: Supplementary file 1 — Supplementary Information [file 41598_2018_27798_MOESM1_ESM.pdf]

*Supplementary Information for:*

# Cracking effects in squashable and stretchable thin metal films on PDMS for flexible microsystems and electronics

Tiffany Baëtens, Emiliano Pallecchi, Vincent Thomy, & Steve Arscott

Institut d'Electronique, de Microélectronique et de Nanotechnologie (IEMN), CNRS, The University of Lille, Cité Scientifique, 59652 Villeneuve d'Ascq, France.

## 1. Thin film roughness and uniformity.

The roughness of evaporated thin films was determined using Atomic Force Microscopy (Dimension D3100 Bruker–Veeco, USA) and analysed using software (Gwyddion).[1] The average roughness and RMS roughness of the evaporated films are shown in Supplementary Table S1. All evaporated films were seen to be uniform by observation of the AFM phase information.

| Material - Thicknesses | Cr –- 0.9nm | Cr – 1.4nm | Cr – 4.1nm | Au – 21.6nm | Cr/Au – 1.4/21.6nm |
|---|---|---|---|---|---|
| **Average roughness: $R_a$** | 0.47 ± 0.13 | 0.38 ± 0.07 | 0.22 ± 0.04 | 0.72 ± 0.29 | 0.43 ± 0.18 |
| **RMS roughness: $R_q$** | 0.57 ± 0.1 | 0.51 ± 0.13 | 0.3 ± 0.12 | 1.01 ± 0.43 | 0.57 ± 0.23 |

**Supplementary Table S1: Summary of AFM results.**

## 2. Effect of different thin metal films evaporated on PDMS.

### *2.1 Effect of metal type on process-induced cracking (PIC) on thin metal films evaporated onto PDMS*

Supplementary Figure S1 shows photographs of evaporated thin films on PDMS immediately after evaporation. The metallization types and thicknesses are gold (21.6 nm) — Fig. 3a, nickel/gold (2.6/21.6 nm) — Fig. 3b, titanium/gold (2.6/21.6 nm) — Fig. 3c, and chromium/gold (2.6/21.6 nm) — Fig. 3d.

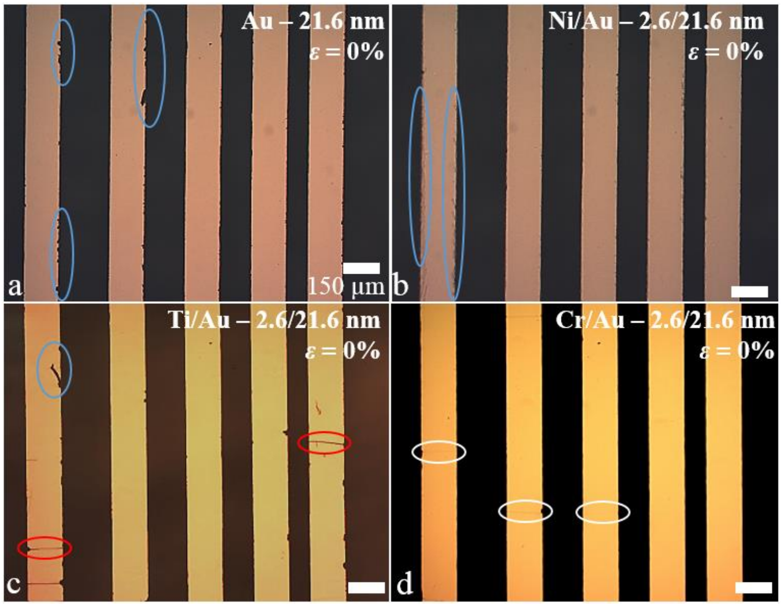


**Supplementary Figure S1: The effect of metal type on process-induced cracking of thin metal films evaporated onto PDMS at zero strain. (a)** Gold lines (21.6 nm) on PDMS. **(b)** Nickel/gold lines on PDMS (2.6/21.6 nm). **(c)** Titanium/gold lines on PDMS (2.6/21.6 nm). **(d)** Chromium/gold lines on PDMS (2.6/21.6 nm). The scale bars are 150µm.

It was observed that there were no PIC when using evaporated gold alone and nickel as an adhesion film—despite several edge defects caused by physical ‘shadow’ masking (indicated by the blue ellipses in Supplementary Figure S1a, Figure S1b and Figure S1c. Such edge defects can cause stress concentration regions which lead to cracking. However, it was noted that gold did not adhere well to PDMS surface (even after an optimised[2] oxygen plasma treatment which all PDMS samples were subjected to prior to evaporation of the metal films)—this is well known[3]. Some PIC was observed when using titanium as the adhesion layer (indicated by the red ellipses in Supplementary Figure S1c although it should be noted that many lines were crack-free. These results indicate that there is little residual tensile stress in these films—this is well documented when evaporating such thin films onto a rigid substrate material.[4,5] Indeed, Bowden *et al.*[6] reported organized ordered structures—presumably not cracked—using such metallizations onto a soft substrate (PDMS). In contrast, when using chromium films, PIC is apparent in most of the lines—see the white ellipses in Figure S1d. The process-induced stresses in chromium thin films are well known to be much higher—at least when using rigid substrates.[4] The spontaneous cracking following thermal evaporation has the characteristic nature of being perpendicular to the line edges—as was previously observed in similarly metallized PDMS systems.[2] The result of the cracking is the formation of isolated rectangular metallic *mesa* features along the lines—which

suggest a certain degree of ordering[2] due to a characteristic crack spacing.[7] This can be contrasted with the ordering in such systems due to buckling.[6]

### *2.2 Effect of metal type on strain-induced cracking (SIC) on thin metal films evaporated onto PDMS*

Supplementary Figure S2 shows examples of post-process, strain-induced cracking for the different metallizations studied—gold (Fig. S2a), nickel/gold (Fig. S2b), titanium/gold (Fig. S2c), and chromium/gold (Fig. S2d). The thickness of the gold was 21.6 nm—the thickness of the adhesion metals was 2.6 nm for clarity of the cracks along the lines at low strains.

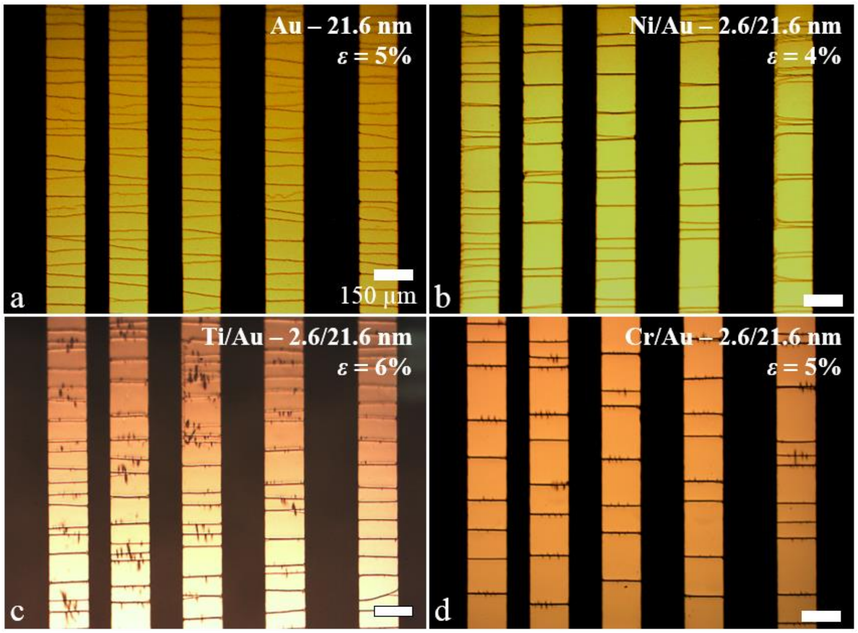


**Supplementary Figure S2. Strain-induced cracking (SIC) for different thin metal films evaporated onto PDMS. (a)** Gold lines (21.6 nm) with no adhesion layer. **(b)** Nickel/gold (2.6/21.6 nm) lines. **(c)** Titanium/gold (2.6/21.6 nm) lines. **(d)** Chromium/gold (2.6/21.6 nm) lines. The applied strain in all cases is between 4% and 6%. The lines are 150 µm wide. The scale bars are 150µm.

Supplementary Figure S2 was obtained by applying uniaxial strain along the longest length of the metal lines. Upon the application of uniaxial strain, SIC was observed in all samples—it was seen that the perpendicularity and periodicity of the SIC depends on the type of metal and its thickness. For example, gold films (in the absence of an adhesion metal) resulted in cracking which was not perpendicular compared to gold adhered to PDMS using a chromium film. When

using nickel and titanium as the adhesion metal, SIC was observed in films that presented no or little PIC following processing. The SIC in such films was observed to occur at very small stresses ~5%.

## 3. Mechanical properties

### *3.1 Bulk mechanical properties of materials used in the study*

| | Poisson coefficient ν | Young's modulus *E* | Tensile strength *uts* |
|---|---|---|---|
| **PDMS (10:1)[8]** | 0.5 | 2.5 MPa | 1.9 MPa |
| **Gold** | 0.4 | 79 GPa | 100-120 MPa |
| **Chromium** | 0.21 | 279 GPa | 282 MPa |
| **Nickel** | 0.31 | 200 GPa | 140-195 MPa |
| **Titanium** | 0.32 | 116 GPa | 246-370 MPa |

**Supplementary Table S2: Bulk values of the mechanical properties of the materials used in the study.**

### *3.2 Failure strain and Young's Modulus of thin chromium films from the literature[2]*

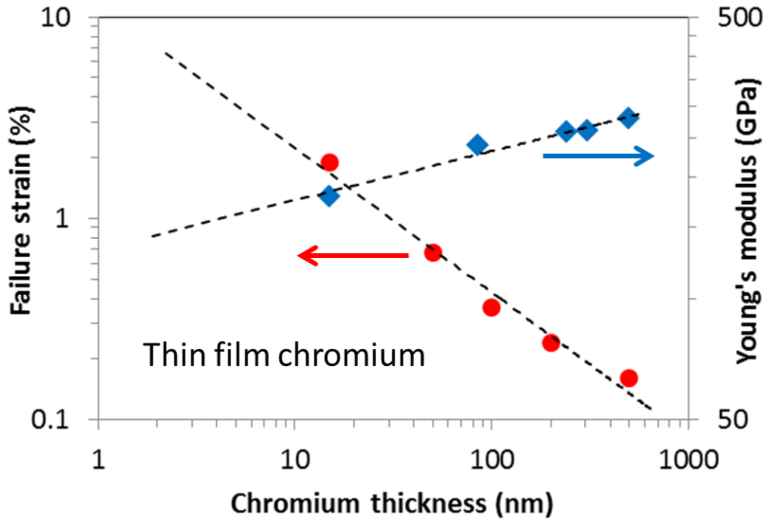


**Supplementary Figure S3: The value of Young's modulus (filled blue triangles) and failure strain (filled red circles) of chromium films versus film thickness. The points are data found in the literature.[2]**

The values of failure strain $\varepsilon_c$ and Young's modulus $E$ as a function of chromium film thickness $t$ given in the literature can be fitted by the following functions:

$$\varepsilon_c = 11.644t^{-0.717}$$

$$E = 132.55t^{0.122}$$

## 4. 3D profile of cracking of the longitudinally orientated metal lines on PDMS.

Supplementary Figure S4 shows 3D optical profiling of the longitudinally orientated metal lines evaporated onto PDMS. The images are obtained using interference microscopy with a Contour GT-X 3D optical profiler (Bruker Corp., USA).

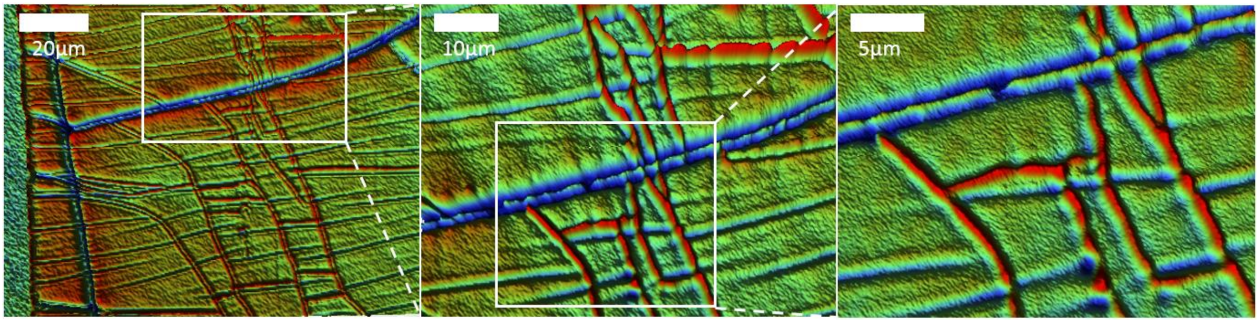


**Supplementary Figure S4: 3D profiles of the longitudinally orientated metal lines on PDMS.**

## 5. Gaussian fit with the experimental data.

The crack spacing data can be tested for *normality*—i.e. its agreement with a normal (Gaussian) distribution. In order to do this, the crack spacing $\lambda$ data of every sample—and at every strain value— ($n$ values) is first sorted from lowest to highest value. Next, each data value (crack spacing $\lambda$ value) is assigned an integer data number $(i)$. Following this, the below formula is implemented to calculate the *z-score* $(z_i)$:

$$z_i = \Phi^{-1}\left(\frac{i - 0.5}{n}\right)$$

where $\Phi^{-1}$ is the *standard normal quantile function*[9] The above equation is valid provided that $n > 10$—this is the case in or work, $n$ is the number of crack spacing. The solution of the above equation can be easily solved using software e.g. Excel 2016 (Microsoft, USA). Finally, the sorted data (crack spacing $\lambda$) is plotted on the abscissa (x axis) versus the corresponding value of $z_i$ on the ordinate (y axis) to give what is known as a *normal probability plot*. Supplementary Figure S5 shows typical normal probability plots obtained from some of our crack spacing data.

Several points can be noted from these plots. First, if the data perfectly followed a normal distribution, the plots would be perfectly linear. Clearly this is not the case, but they do *approximate* a linear trend—this justifies the use of a standard deviation calculation for the *error* in the paper. Secondly, the approximation to a normal distribution seems to improve as mechanical strain is applied and the crack spacing is reduced. This is shown in Supplementary Table S3 where the coefficient of determination ($R^2$) is seen to increase with strain. This latter observation possibly suggests increased ordering in the system at smaller length scales (lower crack spacing) obtained by mechanical strain. Finally, at a higher strain value (10% here) some low values of crack spacing deviate from a normal distribution—see Inset to Supplementary Figure S5). These very well defined crack spacing are linked to measurement resolution. More work needs to be done to elaborate on these observations.

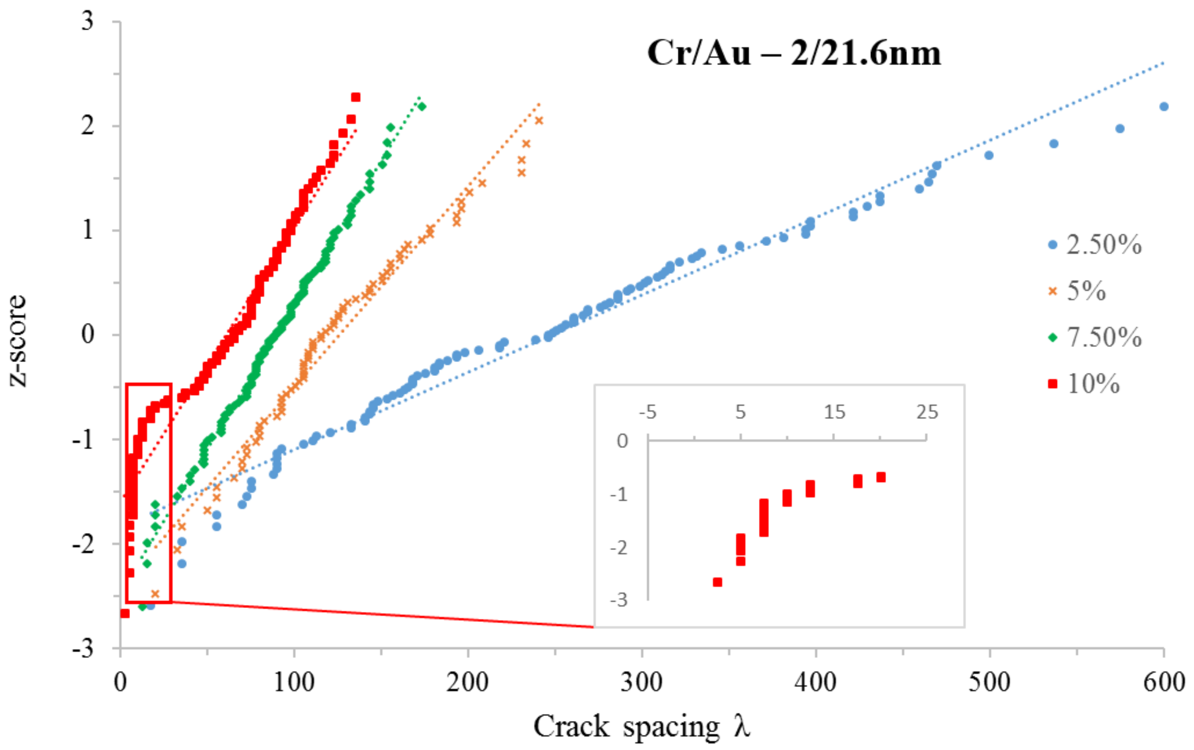


**Supplementary Figure S5: Normal probability plot for Cr/Au.**

Supplementary Table S3 shows the values of the coefficient of determination $(R^2)$ of the normal probability plots shown in Supplementary Figure S5.

| Strain (%) | $R^2$ |
|---|---|
| 2.5 | 0.966 |
| 5 | 0.968 |
| 7.5 | 0.989 |
| 10 | 0.99 |

**Supplementary Table S3: Numerical results of experimental data gathered in the study.**

The data can be fitted to Gaussian functions—these are shown in Supplementary Figure S6 which plots the distribution of the crack spacing.

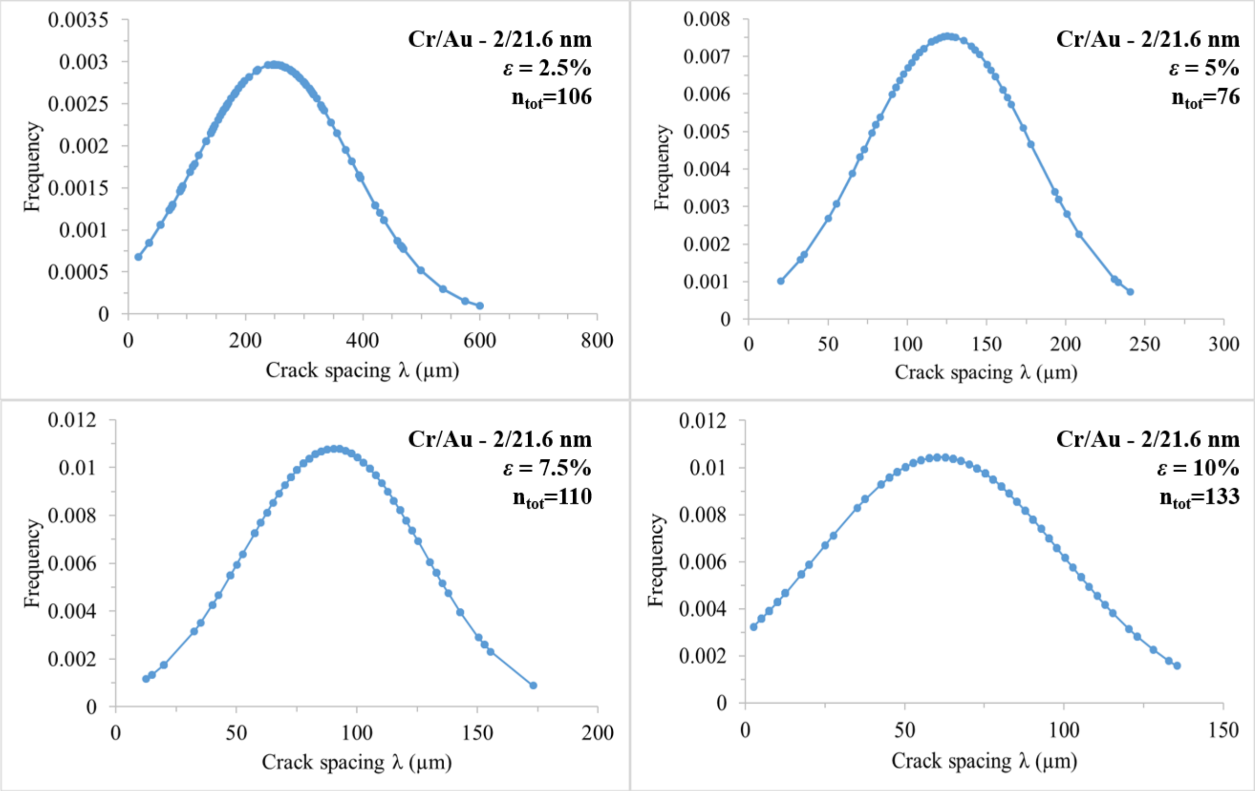


**Supplementary Figure S6: Gaussian plots of the experimental data. $n_{tot}$ represent the number of crack spacing.**

## 6. Tabular values of results

### *6.1 Numerical data of Figure 6 and Figure 7 of the manuscript*

The follow table gives the data gathered in the study in numerical format.

| | Strain (sample) | Crack spacing | | Number of cracks (per length) | |
|---|---|---|---|---|---|
| | | **Lambda (μm)** | **SD** | **N ($mm^{-1}$)** | **SD** |
| Cr/Au (0.9/21.6 nm) | 2.5% | 717.32 | 291.37 | 1.48 | 0.14 |
| | 5% | 196.60 | 54.86 | 4.83 | 0.14 |
| | 7.5% | 142.40 | 46.35 | 7.92 | 0.26 |
| | 10% | 95.59 | 32.25 | 10.79 | 0.23 |
| Cr/Au (1.4/21.6 nm) | 2.5% | 575.11 | 220.04 | 2.08 | 0.04 |
| | 5% | 153.01 | 49.10 | 6.50 | 0.20 |
| | 7.5% | 83.95 | 38.59 | 10.94 | 0.09 |
| | 10% | 62.73 | 18.32 | 15.09 | 0.18 |
| Cr/Au (2/21.6 nm) | 2.5% | 255.86 | 80.54 | 3.72 | 0.19 |
| | 5% | 126.41 | 26.31 | 8.55 | 0.35 |
| | 7.5% | 87.02 | 19.54 | 12.43 | 0.26 |
| | 10% | 59.75 | 19.39 | 15.53 | 0.49 |
| Cr/Au (2.6/21.6 nm) | 2.5% | 368.70 | 139.67 | 2.79 | 0.23 |
| | 5% | 127.22 | 35.27 | 8.48 | 0.30 |
| | 7.5% | 90.15 | 23.06 | 12.07 | 0.18 |
| | 10% | 67.55 | 16.96 | 14.97 | 0.53 |
| Cr/Au (3.3/21.6 nm) | 2.5% | 539.80 | 107.01 | 1.94 | 0.11 |
| | 5% | 331.86 | 90.21 | 3.32 | 0.16 |
| | 7.5% | 240.03 | 53.39 | 4.68 | 0.21 |
| | 10% | 185.08 | 40.47 | 6.44 | 0.22 |

**Supplementary Table S4: Numerical results of experimental data gathered in the study.**

### *6.2 Numerical values of slopes and coefficient of determination of the data presented in Figure 7 of the manuscript.*

The experimental data in Figure 7 of the manuscript is fitted using a linear regression. The equation for the number of cracks $N$ ($mm^{-1}$) is as a function of applied strain $\varepsilon$ (%) is:

$$N = \alpha\varepsilon + \beta$$

The numerical values of the slopes $\alpha$, the intercepts $\beta$, and the coefficient of determination $R^2$ are given in Supplementary Table S5.

| Cr thickness (nm) | Slope α | Intercept β | $R^2$ |
|---|---|---|---|
| **0.9** | 1.241 | -1.497 | 0.998 |
| **1.4** | 1.777 | -2.537 | 0.999 |
| **2** | 1.572 | 0.233 | 0.991 |
| **2.6** | 1.605 | -0.453 | 0.975 |
| **3.3** | 0.595 | 0.377 | 0.996 |

**Supplementary Table S5: Numerical values of the slopes, intercepts, and the coefficient of determination $R^2$ for the data presented in Figure 7 of the manuscript.**

## 7. Plot of the coefficient of variation

In an effort to reveal an underlying ordering in the strain-induced cracking the ratio of the dispersion (standard deviation) to the average crack spacing can be plotted as a function of strain for several chromium thicknesses. The ratio of the standard deviation to the average is known as the coefficient of variation (CV)—which in this case we can use to measure order (here the crack spacing regularity) as the scale (here the crack length) changes. For the chromium/gold films this is plotted in Supplementary Figure S7. There are two main observations here: first, independent of the thickness, increasing the strain is seen to lead to an increase in the apparent ordering of the cracking. Second, at higher strains, decreasing the chromium thickness leads to an apparent increase in the ordering of the resulting cracking. For example, at a strain value of 7.5% decreasing the chromium thickness from 3.3 nm to 1.4 nm results in a decrease of the CV of >5. One can suggest tentative explanations for this. In terms of the strain relationship, at low strains the influence of edge defects, and their associated stress concentration, leads to initially random or 'less periodic' cracking. As the strain is increased, the cracking becomes dominated by the periodic cracking predicted by modelling of perfect layers. In terms of the chromium thickness, at lower strains the experimental results suggest a randomness which agrees with the latter argument concerning edge defects. At higher strains, thinner films seem to result in higher regularity of cracking—at the moment we cannot explain this. Although, in principle, if thinner films become non-uniform, possible random micro-cracking in the layer may lead to a reduction of the cracking ordering—but this is not in agreement with the observations. This observation requires more investigation.

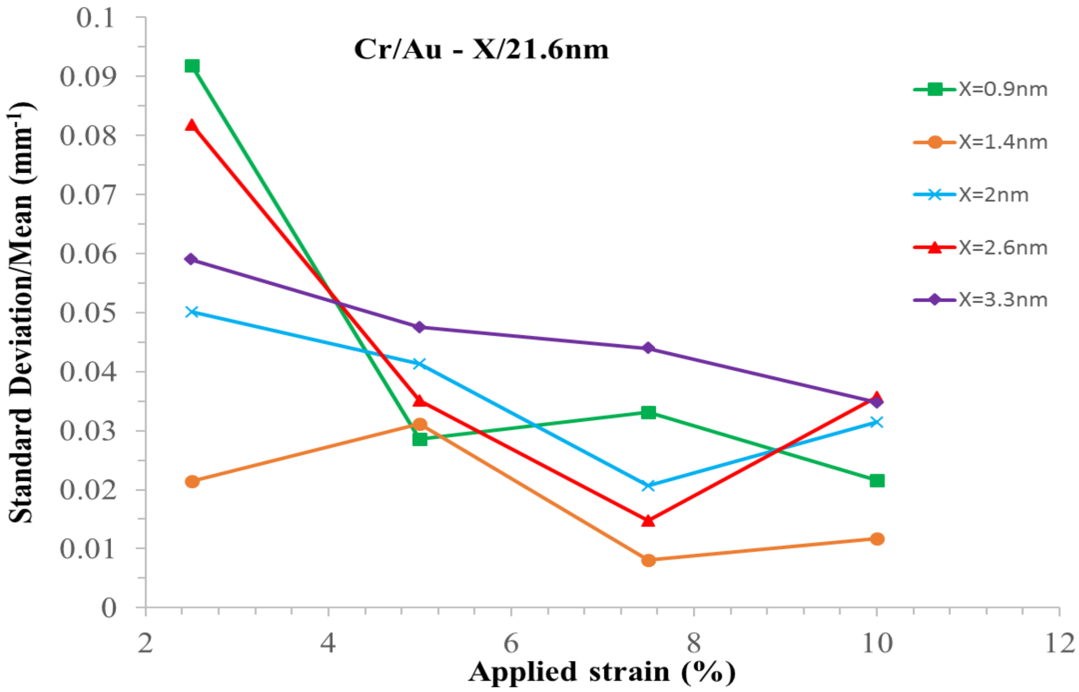


**Supplementary Figure S7: A plot of the error/average crack spacing ratio as a function of applied strain and chromium thickness.** Five different chromium film thicknesses ranging from 0.9 nm to 3.3 nm are shown. The data was gathered using metal lines 7.95 mm by 150 µm on PDMS.

## 8. Plot of crack density versus chromium thickness and strain

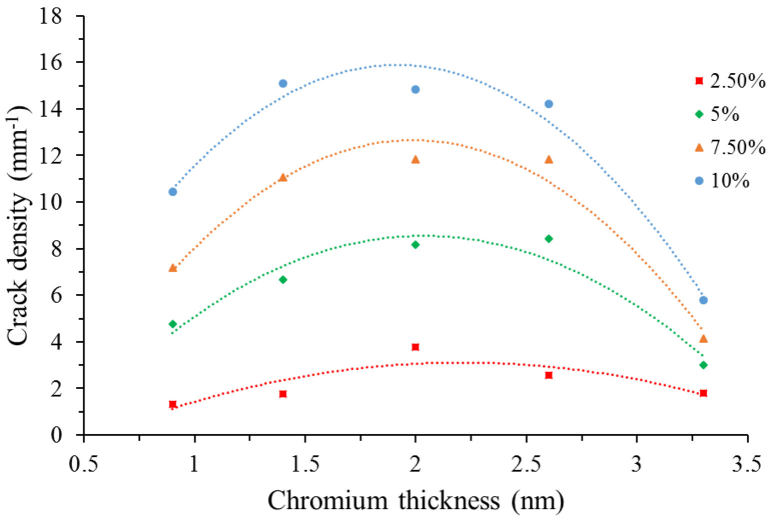


**Supplementary Figure S8: A plot of the crack density as a function of applied strain and chromium thickness.** Five different chromium film thicknesses ranging from 0.9nm to 3.3nm.

## 9. Plot of crack spacing taking into account the extrapolated variation of failure strain and Young's modulus

Supplementary Figure S9 shows a calculation of the crack spacing (based on the equation given in the manuscript[7,10]). Supplementary Figure S9a considers a constant value of $\Gamma_f$—Supplementary Figure S9b considers that $\Gamma_f$ varies linearly with the experimentally observed values of ultimate tensile strength available in the literature (see Supplementary Figure S3).

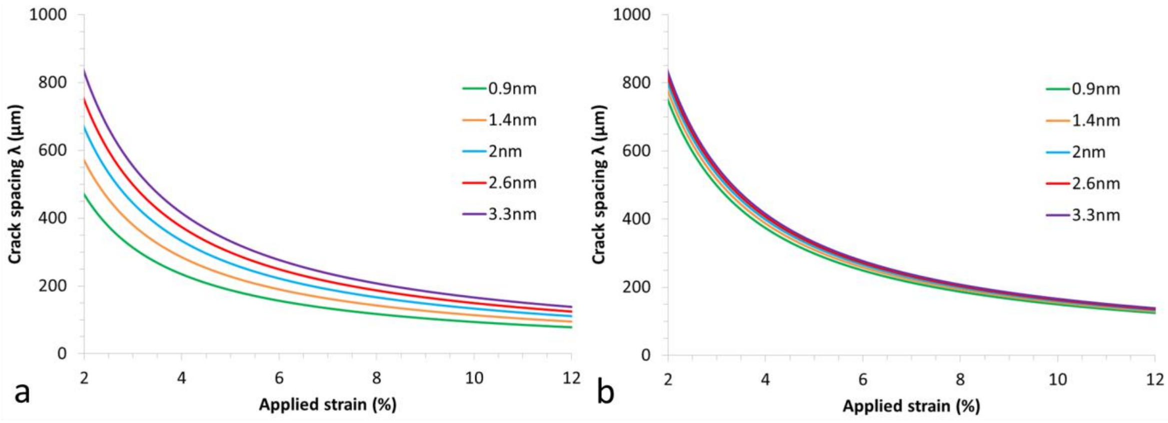


**Supplementary Figure S9: The cracking spacing λ of a thin film subjected to strain ε. (a)** Accounting for the change in Young's modulus (*E*)—with the work of fracture ($\Gamma_f$) fixed. **(b)** Accounting for a change in both E and $\Gamma_f$.

## 10. Measured fracture toughness of the thin films

| | **Fracture toughness $K_{Ic}$ MPa $\sqrt{m}$** |
|---|---|
| **Au (21.6 nm)** | 0.1 |
| **Cr/Au (0.9/21.6 nm)** | 13 ± 6 |
| **Cr/Au (1.4/21.6 nm)** | 5 ± 4 |
| **Cr/Au (2/21.6 nm)** | 4 ± 2.5 |
| **Cr/Au (2.6/21.6 nm)** | 4.5 ± 4 |
| **Cr/Au (3.3/21.6 nm)** | 10.5 ± 4.6 |
| **Ti/Au (2.6/21.6 nm)** | 1.4 |
| **Ni/Au (2.6/21.6 nm)** | 1.5 |

**Supplementary Table S6: Values of the fracture toughness extracted from the experimental results presented in Supplementary Fig. S2 and Fig. 7.**

## 11. Electrical characterization for transversally orientated metal lines

*11.1 Optical micrograph photographs of the probes used for the electrical characterization.*

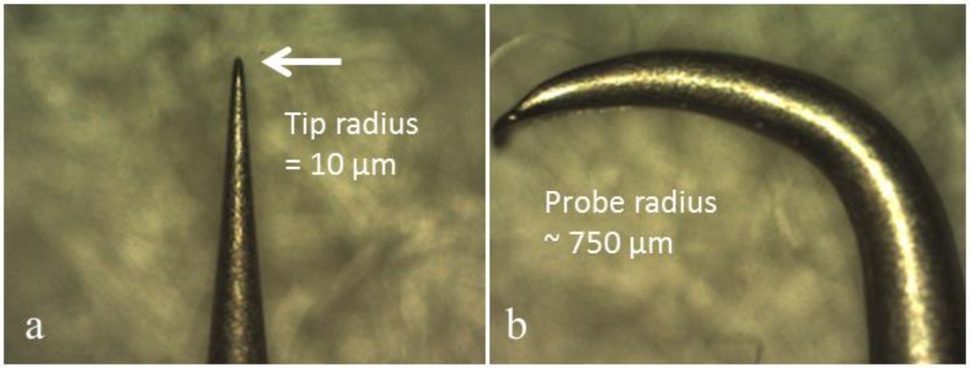


**Supplementary Figure S10: Optical micrograph photographs of the probes used for the electrical characterization. (a)** Small tipped probes. **(b)** Large tipped probes.

*11.2 3D and 2D profile of cracking*

Supplementary Figure S11 show 2D and 3D optical profiling of the metal lines evaporated onto PDMS. The images are obtained using interference microscopy with a Contour GT-X 3D optical profiler (Bruker Corp., USA).

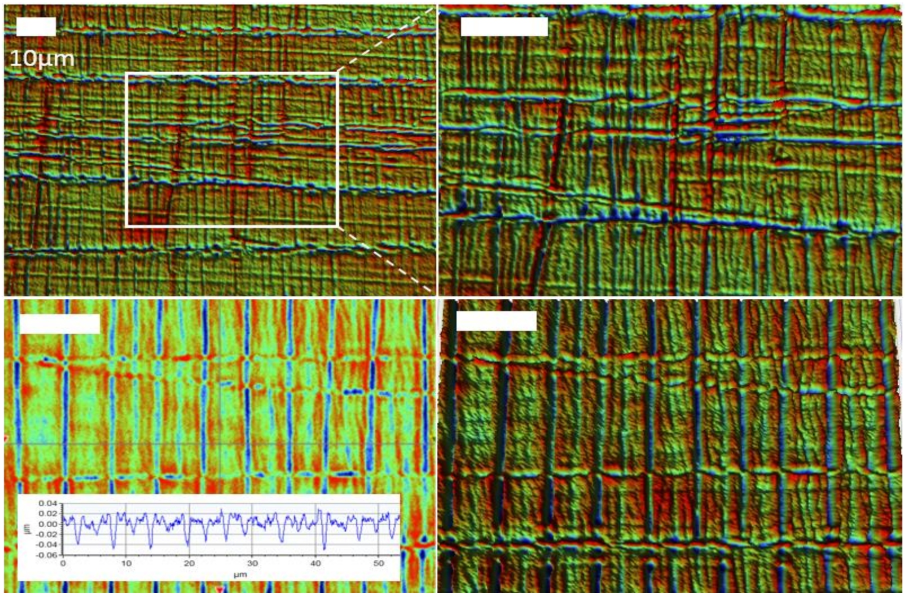


**Supplementary Figure S11: 3D and 2D profiles of transversally orientated metal lines on PDMS.** The scale bars are 10µm.

*11.3 Typical current-voltage curves of transversally orientated metal lines on PDMS*

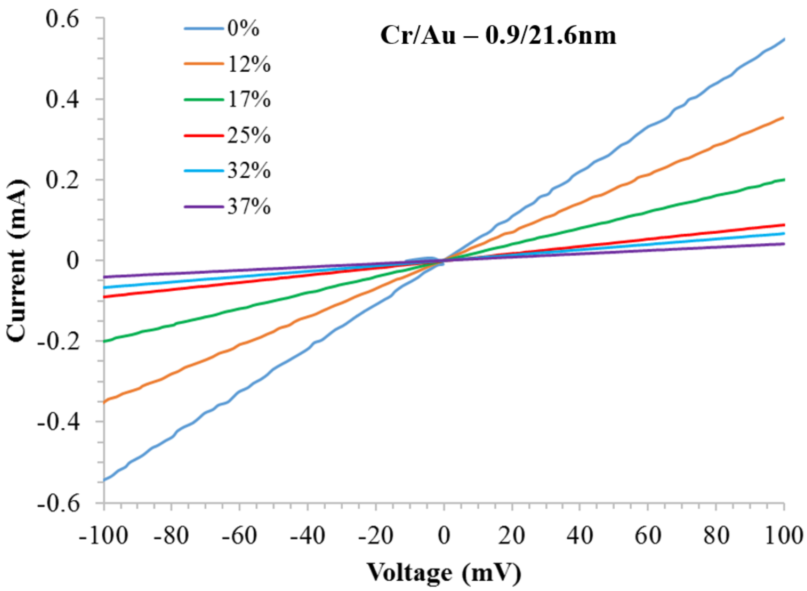


**Supplementary Figure S12: Current-voltage characteristics of transversally orientated metal lines (Cr/Au) on PDMS supports as a function of applied strain (%).**

## 12. Derivation of gauge factor formulae for a thin film metal line in the small strain limit.

In the following derivations, the strain $\varepsilon$ is the strain in the metal film—referred to as $\varepsilon_f$ in the manuscript. The piezoresistive gauge factor consider lines that have a length $L$, a width $w$, and a thickness $t$—where $L$>>$w$ and $t$. The following equations consider uniquely a metal film and its associated strain $\varepsilon$ due to mechanical stress. The electrical resistance is measured *along* $L$ in each case. The electrical resistivity of the metal is $\rho$—the Poisson coefficient of the metal film is $\nu$. The gauge factor is derived for two cases: lines orientated parallel to the longitudinally strain and orientated perpendicular to the applied longitudinal strain—see Supplementary Figure S13.

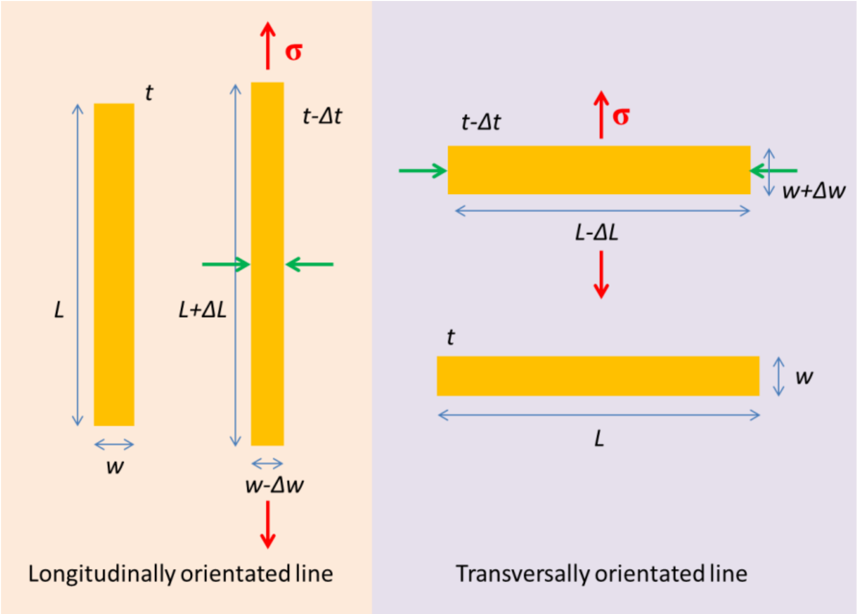


**Supplementary Figure S13: Orientation of metal lines. Left image—longitudinally orientated lines. Right image—transversally orientated image.**

*12.1 Lines orientated parallel to the longitudinal strain—Longitudinal gauge factor $GF_L$*

The zero-strain electrical resistance of the line is given by:

$$R = \frac{\rho L}{wt}$$

Assuming no cracking, the strained resistance $R_s = R + \Delta R$—along the length $L$—due purely geometrical changes is given by:

$$R_s = \frac{\rho(L + \Delta L)}{(w - \Delta w)(t - \Delta t)}$$

by inserting the strain $\varepsilon$ and the Poisson coefficient $\nu$ we have:

$$R_s = \frac{\rho(L + \varepsilon L)}{(w - \nu\varepsilon w)(t - \nu\varepsilon t)}$$

$$R_s = \frac{\rho L(1 + \varepsilon)}{wt(1 - \nu\varepsilon)^2}$$

The gauge factor is defined as:

$$GF = \frac{\Delta R}{R} \Big/ \varepsilon$$

Therefore the *longitudinal* gauge factor of the line, where the resistance is measured along the length $L$, can be written as:

$$GF_L = -\frac{1 + 2\nu - \nu^2\varepsilon}{(\nu\varepsilon - 1)^2}$$

### *12.2 Lines orientated perpendicular to the longitudinal strain—Transverse gauge factor $GF_T$*

In order to obtain an expression for the *transverse* gauge factor, we can apply the same logic as above except in this case the width $w$ is stretched whilst the length $L$ and thickness $t$ of the lines are compressed due to the Poisson effect. Thus we can write down the following expression for the strained resistance $R_s = R + \Delta R$ along $L$ as:

$$R_s = \frac{\rho(L - \Delta L)}{(w + \Delta w)(t - \Delta t)}$$

Again by inserting the strain $\varepsilon$ and the Poisson coefficient $\nu$ we have:

$$R_s = \frac{\rho(L - \nu\varepsilon L)}{(w + \varepsilon w)(t - \nu\varepsilon t)}$$

$$R_s = \frac{\rho L(1 - \nu\varepsilon)}{wt(1 + \varepsilon)(1 - \nu\varepsilon)}$$

$$R_s = \frac{\rho L}{wt}\frac{1}{(1 + \varepsilon)}$$

As above, the gauge factor is defined as:

$$GF = \frac{\Delta R}{R} \Big/ \varepsilon$$

Therefore the *transverse* gauge factor of the line, where the resistance is measured along the length $L$, can be written:

$$GF_T = -\frac{1}{(1+\varepsilon)}$$

Note that the longitudinal gauge factor is dependent on the Poisson coefficient i.e. a mechanical property of the material whereas the transverse gauge factor is only dependent on the applied strain. In other words, for a transversally orientated gauge the same gauge factor would be obtained for any conducting material having the same dimensions.

## Supplementary References


1. Nečas, D. & Klapetek, P. Gwyddion: an open-source software for SPM data analysis. *Open Phys.* **10,** (2012).
2. Seghir, R. & Arscott, S. Controlled mud-crack patterning and self-organized cracking of polydimethylsiloxane elastomer surfaces. *Sci. Rep.* **5,** 14787 (2015).
3. Ferguson, G. S., Chaudhury, M. K., Sigal, G. B. & Whitesides, G. M. Contact Adhesion of Thin Gold Films on Elastomeric Supports: Cold Welding Under Ambient Conditions. *Science* **253,** 776–778 (1991).
4. Klokholm, E. & Berry, B. S. Intrinsic Stress in Evaporated Metal Films. *J. Electrochem. Soc.* **115,** 823–826 (1968).
5. Thornton, J. A. & Hoffman, D. W. Stress-related effects in thin films. *Thin Solid Films* **171,** 5–31 (1989).
6. Bowden, N., Brittain, S., Evans, A. G., Hutchinson, J. W. & Whitesides, G. M. Spontaneous formation of ordered structures in thin films of metals supported on an elastomeric polymer. *Nature* **393,** 146–149 (1998).
7. Thouless, M. D., Li, Z., Douville, N. J. & Takayama, S. Periodic cracking of films supported on compliant substrates. *J. Mech. Phys. Solids* **59,** 1927–1937 (2011).

8. Seghir, R. & Arscott, S. Extended PDMS stiffness range for flexible systems. *Sens. Actuat A* **230,** 33–39 (2015).
9. Chambers, J. M., Cleveland, W. S., Kleiner, B. & Tukey, P. A. *Graphical methods for data analysis*. (Duxbury Press, 1983).
10. Thouless, M. D. Crack Spacing in Brittle Films on Elastic Substrates. *J. Am. Ceram. Soc.* **73,** 2144–2146 (1990).
